# The geographic, environmental and phylogenetic evolution of the Alveolinoidea from the Cretaceous to the present day

**DOI:** 10.14324/111.444/ucloe.000015

**Published:** 2021-03-08

**Authors:** Marcelle K. BouDagher-Fadel, Geoffrey David Price

**Affiliations:** 1 Office of the Vice-Provost (Research), University College London, 2 Taviton Street, London WC1H 0BT, UK

**Keywords:** foraminifera, alveolinoids, Cretaceous, Paleogene, Neogene, Holocene, biostratigraphy, phylogeny, palaeoenvironment, palaeogeographic distribution, extinction, evolution, sea-level change, homoplasy, the environment, climate, ecology

## Abstract

The superfamily Alveolinoidea is a member of the Order Miliolida, and comprises three main families, the Alveolinidae, the Fabulariidae and the Rhapydioninidae. They are examples of Larger benthic foraminifera (LBF), which are single-celled organisms with specific characteristic endoskeletons. Alveolinoids are found globally from the Cretaceous to the present day, and are important biostratigraphic index fossils in shallow-marine carbonates. They are often associated with hydrocarbon reservoirs, and exhibit provincialism with characteristic genera often confined to one of the American, Tethyan or Indo-Pacific provinces. Previously, the systematic study of the global interrelationship between the various alveolinoid lineages has not been possible because of the absence of biostratigraphic correlation between the geographically scattered assemblages, and the scarcity of described material from the Indo-Pacific province. Here we use the literature and new material from the Americas, the French Alps, Iran, Tibet, India and South East Asia, coupled with the use of the planktonic foraminiferal zonal (PZ) correlation scheme to propose a comprehensive, global, systematic analysis of the biostratigraphic, phylogenetic and paleogeographic evolution of the alveolinoids. The alveolinoids originated in the Cretaceous in the Tethyan province. During a global sea-level low stand, a westward migration of some alveolinoids species to the Americas occurred, a behaviour previously reported in contemporaneous orbitolinid LBF. After the Cretaceous/Palaeogene (K–P) event, which saw the extinction of all Cretaceous alveolinoids, rare new forms of alveolinoids evolved again, first in the Americas and later independently in Tethys. As was found in previous studies of rotalid LBF, sea-level low stands in the Paleocene also allowed some alveolinoid forms to migrate, but this time in an eastward direction from the Americas to Tethys, and from Tethys on to the Indo-Pacific province. Alveolinoids still exist today (*Borelis* and *Alveolinella*), the former of which is cosmopolitan, while the latter is restricted to the Indo-Pacific province. Throughout their phylogenetic history, alveolinoids characteristically exhibit convergent evolution, with the repeated re-occurrence of certain morphological features. Understanding this propensity to homoplasy is essential in understanding and constructing the phylogenetic relationships within the alveolinoid superfamily.

## Introduction

Larger benthic foraminifera (LBF) are single-celled organisms, with an internal, characteristic test, that have acted as major reef-forming organisms from the Paleozoic to the present day (see [1]). Within the geological record, there are 14 taxonomically defined orders of LBF. They are widely used as key biostratigraphic and palaeoenvironmental indicators. Although they were generally limited to warmer, shallower marine environments, their fossil forms are globally distributed, but frequently exhibit provincialism at the family or generic level.

Our previous work has established that global marine regressions correlate with the migration of some LBF genera from one province to another. The inter-provincial migration of a genus is characterised and defined by the contemporaneous occurrence of the same species (although they may have been given different names, which are therefore synonyms) in two previously isolated provinces. In the Cretaceous, for example, we inferred that, during a global sea-level low stand, some orbitolinids (agglutinated foraminifera, members of the Order Textulariida) underwent a westward trans-oceanic migration from their Tethyan province of origin to the American province (see [2]). Although subsequently the Tethyan province remained the hotspot for orbitolinid speciation, parallel lineages evolved in the again isolated American province, but were extremely rare. Later in the Cenozoic, the American province became the hot spot for speciation of other LBF families (including members of the Order Rotaliida, studied previously by BouDagher-Fadel and Price [3–6]). Again during global sea-level regressions, trans-Atlantic migrations occurred, but in these cases in an eastward direction into Tethys and then on to the Indo-Pacific, and at times to southern Africa.

These previous studies, therefore, have led to the hypothesis that periodic, sea-level low stand enabled, inter-provincial migration is a characteristic global dispersal mechanism of many LBF families. As suggested by Pignatti [7] however, this hypothesis needs further testing. In this study, therefore, we investigate the phylogenetic evolution and palaeogeographic distribution of the main genera of the porcelaneous Alveolinoidea superfamily (members of the Order Miliolida), to establish whether they too exhibit an evolutionary and dispersal behaviour similar to the previously studied LBF groups.

The Alveolinoidea comprise three families, the Alveolinidae, the Fabulariidae and the Rhapydioninidae (see [1]). They range from the Cretaceous to the present day. Throughout their evolutionary lineages, the alveolinoids exhibited relatively rapid rates of evolution and developed complex tests, which make them an important biostratigraphic index fossil group for the shallow-marine environments of the later Mesozoic and the Cenozoic. Today, alveolinoid-bearing limestones occur globally from the Americas, through the Mediterranean and the Gulf, Tibet, and on to the Indo-Pacific province. They are often associated with significant hydrocarbon reservoirs. The study of the Alveolinoidea and the definition of their stratigraphic ranges has been the subject of many regional micropaleontological investigations, especially those of the Tethyan and American provinces [1,8–22], but the most recent revisions of *Alveolina* from Indonesia are by Bakx [23]. Until now, however, it has not been possible to develop an effective global view of their evolution and inter-relationships, because the systematic study of the links between the American, Tethys and Indo-Pacific lineages has been hampered by the lack of biostratigraphic correlation between the geographically scattered alveolinoid assemblages described in the literature, and by the scarcity of described material from the Indo-Pacific province.

**Figure 1:**
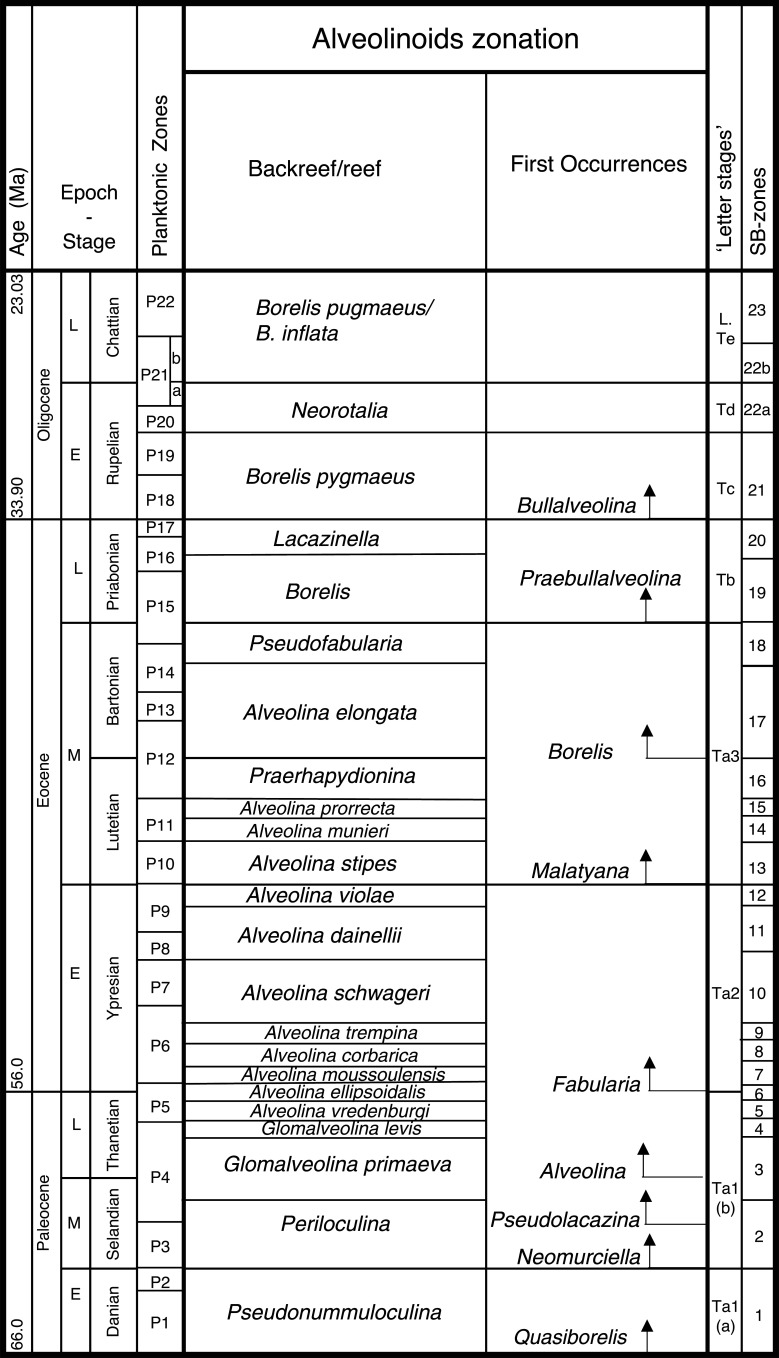
Alveolinoids Paleogene biozones with diagnostic first and last occurrences, calibrated against the PZ, the ‘letter stages’ of the Far East, and the ‘SB’ zones of the Mediterranean, modified after BouDagher-Fadel [1].

**Figure 2:**
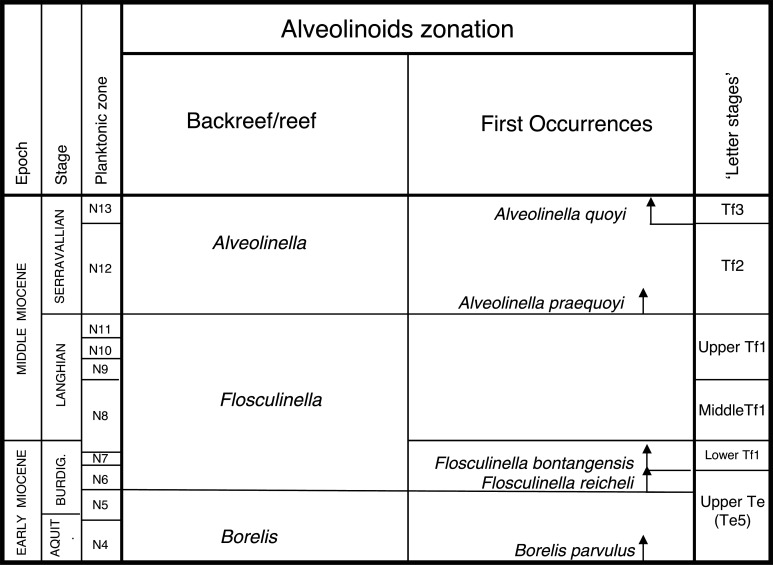
Alveolinoids Neogene biozones with diagnostic first and last occurrences, calibrated to the ‘letter stages’ of the Far East.

In this paper, we revise some of the analysis presented in BouDagher-Fadel [1], and present results from material recently obtained as a result of various investigations of LBF-bearing carbonate facies from two Atlantic basins, one from offshore Brazil (the Campos Basin), and the other off Venezuela (the Matacaibo Basin). These are augmented by studies of new material from several localities including from Saint Barthélemy (Lesser Antilles); Cardenas, San Luis Potosi, Mexico; the shallow marine sequences of the Ainsa Basin, Spain; Iles Madam and Audignon, France; Les Scaffarels sections from the Western Alps; the Circum-Troodos Massif sedimentary succession, northern Cyprus; the island of Paxos, Greece; Latakia, northwest Syria; Lebanon; the Kuh-I-Bingistan section and Savark formation, Iran; the Red Sea; the Pila Spi formations in Jabal Hiebat Sultan, Iraq; the Ladakh region, India; the Tingri and Gamba regions of southern Tibet; the Sekau formation, Indonesia; Port Moresby, New Guinea; the Sarawak Basin (onshore carbonates from Tinjar Province); and the Baden-Brickyard, Baden, Australia. The detailed locations of the materials studied can be found in associated cited references below. As a result, by combining our observations with those in the literature, we are able to propose a comprehensive, global, systematic analysis of the biostratigraphic, phylogenetic and palaeogeographic evolution of the alveolinoids. In our definitions of stratigraphic ranges, we primarily use the planktonic foraminiferal zonal (PZ) scheme of BouDagher-Fadel [24,25], which is tied to the time scale of Gradstein et al. [26]. In this paper, the PZ scheme is also correlated with the LBF ‘letter stages’ of the Far East, as defined by BouDagher-Fadel and Banner [27], and later revised by BouDagher-Fadel [1] (see Figs 1 and 2), and with the biogeographic zonation of shallow benthic larger foraminifera as defined by Serra-Kiel et al. [16], which incorporates a series of biozones proposed for the Paleogene of Western Tethys based on species of *Alvceolina, Nummulites* and *Assilina* [28]. These biozones have been integrated into the shallow benthic zones (SBZ) covering the Paleocene to Eocene of the Mediterranean region by Serra-Kiel et al. [16], as a system of numbered units for the ‘Tertiary’, with SBZ 1–23 covering the Paleogene (see Fig. 1).

As described below, our analysis of the evolution and distribution of the alveolinoids will show that our previously developed hypothesis (i.e. that periodic, sea-level low stands enable inter-provincial migration of LBF [2,7]) is again strongly supported, and that the behaviour and directions of migration of the alveolinoids is resonant with those of the orbitolinids [2], the orthophragminids [6], the nummulitoids [5], the miogypsinids [4] and the lepidocyclinids [3].

The alveolinoids originated in the Cretaceous in the Tethyan province. We conclude here for the first time that during a global sea-level low stand, migration of some alveolinoid species to the Americas occurred. As this province again became isolated as sea levels rose, parallel evolutionary trends between related forms of alveolinoids in the two re-isolated provinces subsequently occurred. After the end-Maastrichtian mass extinction, rare new forms of alveolinoids reappeared first in the Americas. Previous studies also suggested that the Americas were the region of the post-K–P appearance of the rotaliid LBF. As also previously inferred from our studies of a number of rotaliid LBFs, sea-level low stands in the Paleocene allowed some alveolinoid forms to migrate from the Americas to Tethys, where again later sea-level rises led to provincial isolation and parallel provincial evolutionary trends. As also occurred for the previously studied rotaliids, the alveolinoids flourished in Tethys and subsequently migrated from Tethys to the Indo-Pacific province, where they further diversified. With the closure of the Tethys seaway in the Miocene (late Burdigalian), the Mediterranean alveolinoids became isolated from their Indo-Pacific descendants and exhibited parallel evolutionary trends with more evolved, different lineages in the Indo-Pacific province showing morphological convergent trends similar to previous Cretaceous stock. Alveolinoids still exist today (*Borelis* and *Alveolinella*), the former of which is cosmopolitan, while the latter is restricted to the Indo-Pacific province.

The Alveolinoidea throughout their phylogenetic history evolved morphologically divergent features, giving rise to identifiably distinct genera. As in previous biogeographical studies, however, we also confirm spatial and temporal gaps in their phylogenetic/stratigraphic distributions [29–32]. We find that these gaps are best explained by homoplasy, in which convergent and characteristic features reappear as a result of either similar ecological evolutionary selection pressures, or a genetic predisposition to the repetition of specific mutations that produce morphological characteristics that are used to define a genus.

## Morphological and paleoenvironmental characteristics of the Alveolinoidea

**Figure 3:**
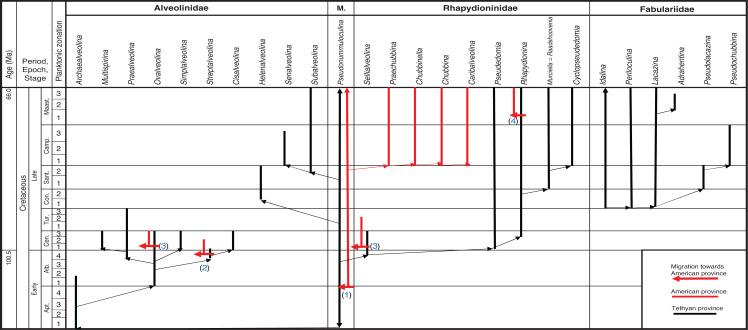
Ranges of the main genera of the Cretaceous Alveolinoidea in the three provinces.

**Figure 4:**
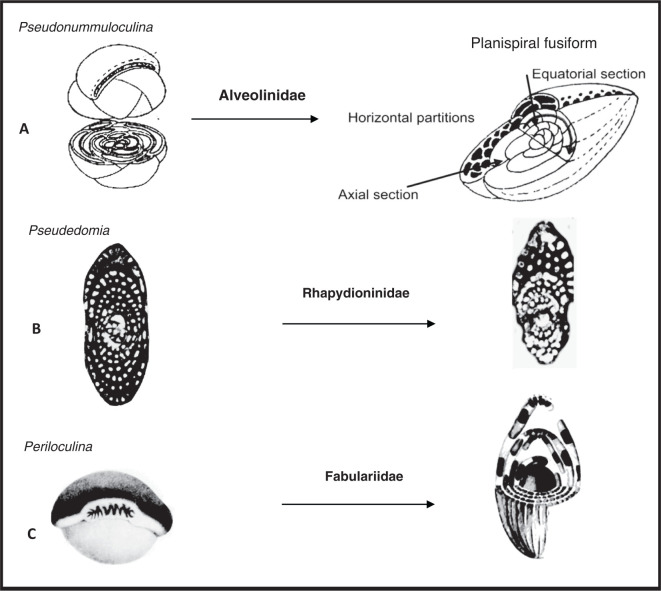
A) The evolution of the Alveolinidae from a simple streptospiral origin, such as *Pseudonummuloculina*; B) The evolution of the Rhapydioninidae from the lenticular compressed *Pseudedomia;* C) The evolution of the Fabulariidae from a completely overlapping form with vertical incomplete partitions, *Periloculina*.

**Figure 5:**
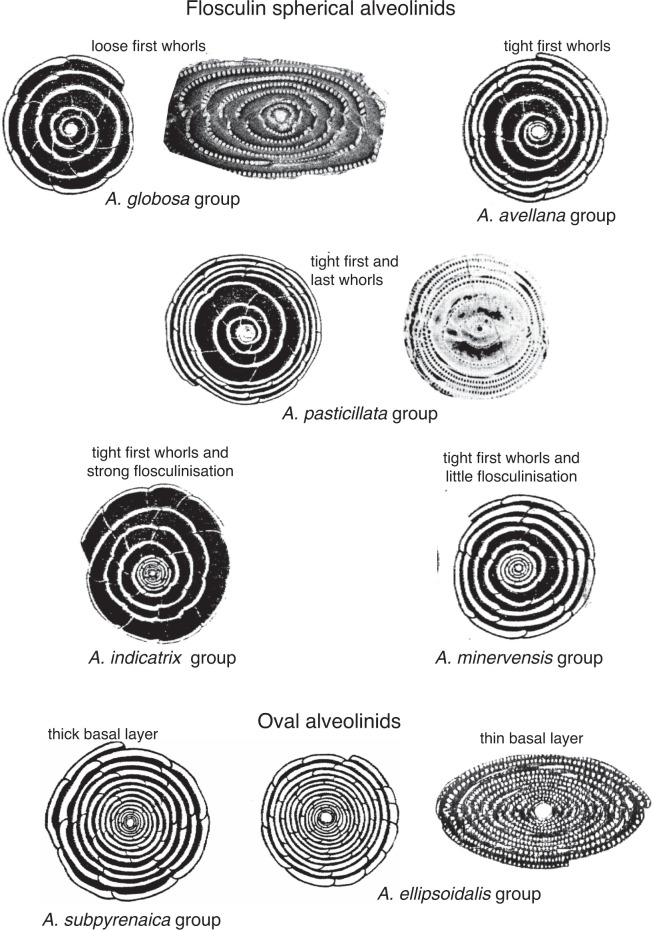
Basal layers and flosculinisation in *Alveolina* (modified from [8] and [1]).

**Figure 6:**
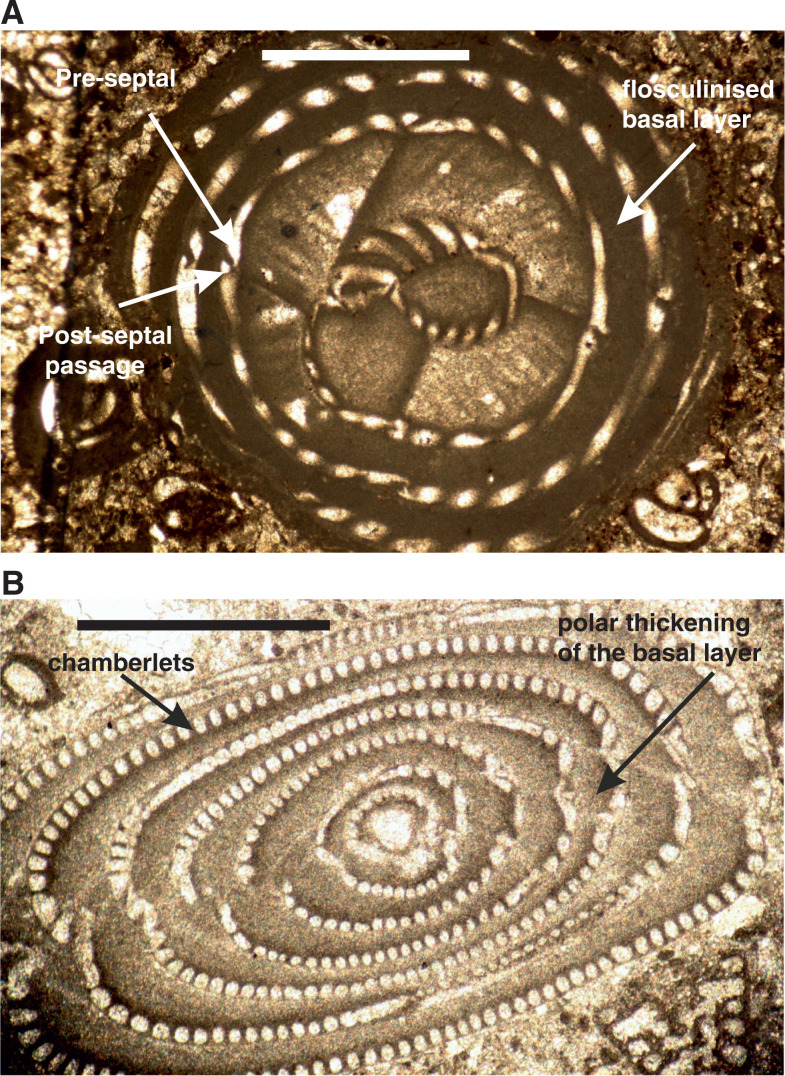
*Alveolina globosa* (Leymerie), A) equatorial section, B) axial section. Early Eocene, Meting Limestone, Laki group, Pakistan, UCL coll. Scale bars = 1 mm.

**Figure 7:**
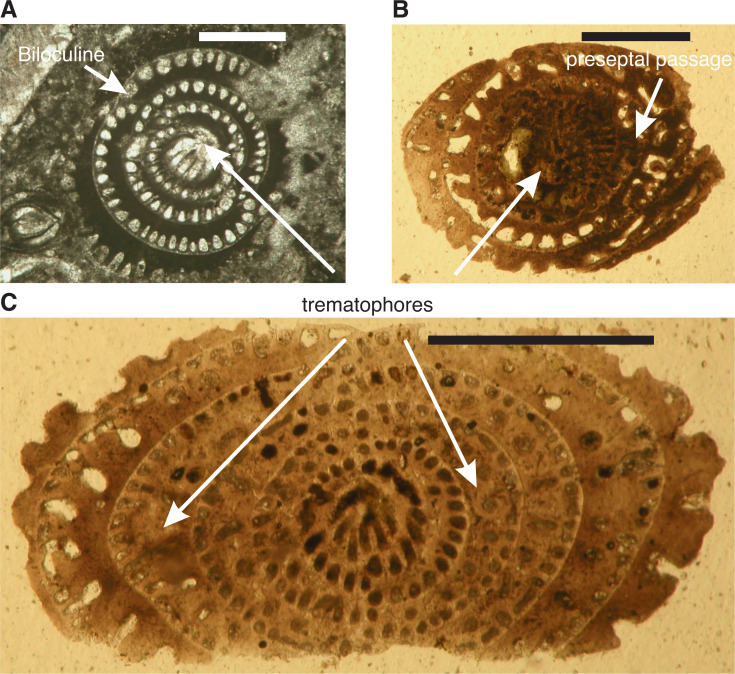
A) *Fabularia hanzawai* Robinson, co-type, Eocene, Saint Andrew Claremont Formation, Jamaica, NHM P52840; B–C) *Fabularia discolithus* Defrance, Lutetian middle calcaire grossier, Chaumont-en-Vexin, Paris Basin, NHM P33071. Scale bars: A) = 0.5 mm; B–C) = 1 mm

The Alveolinoidea made their first appearance in the Early Cretaceous (Fig. 3) exhibiting a fusiform morphology. As is characteristic of miliolids, they have a porcelaneous, imperforate wall structure, with fine, randomly oriented crystals of high-magnesium calcite. Their tests are enrolled along an elongate axis, being initially planispiral or streptospiral, or ‘miliolid’, with chambers added in varying planes (Fig. 4). In an extreme example of ecologically driven convergent evolution involving forms from different orders, the external appearance of alveolinoids closely resembles the planispiral, fusiform fusulinides of the late Paleozoic. This example of homoplasy is the result of ecological selection pressures acting on forms from different orders filling equivalent niches but in different stratigraphic periods. Many alveolinoids attained the same centrimetric scale as the fusulinides, but differ fundamentally in their imperforate, porcelaneous wall structure, and are indeed very distinct when studied in axial and equatorial (median) sections (see [1]). The basic morphology of the alveolinoids is shown in Figs 4–7. Unlike the fusulinides, septal folding does not occur and the spiral septula reach the floor across the chamber. Deposition of secondary calcite (flosculinisation, Figs 4–6) occurs more evenly across the floor of the chamber or may be restricted to a specific period of ontogenesis, rather than being almost entirely concentrated in the axial zone as in the fusulinides. The embryonic apparatus consists of a spherical proloculus followed by a spiral tube (flexostyle), succeeded in some genera by a miliolid nepion (pre-adult stage). In the adult, numerous chambers are planispirally coiled around an elongate axis, each chamber being broken into tubular chamberlets by secondary septa (septula) that run in the direction of coiling. Connectivity between the chambers is enabled through the vertical septula (canals/passages). These may be situated in front of the septum (post-septal) or behind it (pre-septal) (see [1]).

**Figure 8:**
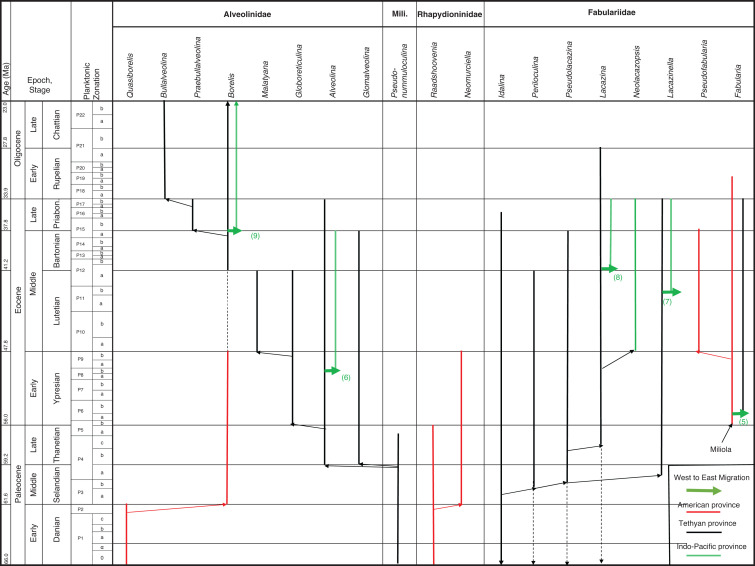
Ranges of the main genera of the Paleogene Alveolinoidea in the three provinces.

In the Cenozoic, the alveolinoids re-appeared in the Paleocene seemingly independently from their Cretaceous relatives (Fig. 8), but evolving from a common simple, long-ranging miliolid ancestor, in an example of homoplasy driven by a predisposition towards the occurrence of a repeated set of mutations that produce generic characteristics. The Paleocene forms share morphological characteristics with their Cretaceous relatives, and evolved separate parallel lineages in both the Tethyan province and the American province. As will be discussed in detail below, this repeated evolution from a simple miliolid common ancestor, such as *Pseudonummuloculina* caused homoplasic generic characteristics, such as alveoles, to reappear on numerous occasions in the Paleogene and in the Neogene.

In the Middle Eocene, alveolinoids reached morphologically large sizes (e.g. *Alveolina prorrecta* Hottinger) growing up to 3.5 cm in the diameter of the axial section [8]. Their sizes were made achievable as a result of the evolutionary development of a variety of morphological structures that had the effect of mechanically strengthening the alveolinoid test, such as by the thickened basal layer (which is subdivided by pillars or secondary partitions in the Fabulariidae), the canaliculation and thickening of the central zone except for a pre-septal space with buttresses or residual pillars (as in the Rhapydioninidae), and the flosculinisation with thick deposition of secondary calcite (as in the Alveolinidae [1]). The basal layer of the larger foraminiferal test, when thickened by flosculinisation [8] or perforated by canalicular passages [33], exists only in the porcelaneous forms (Figs 5–7).

**Figure 9:**
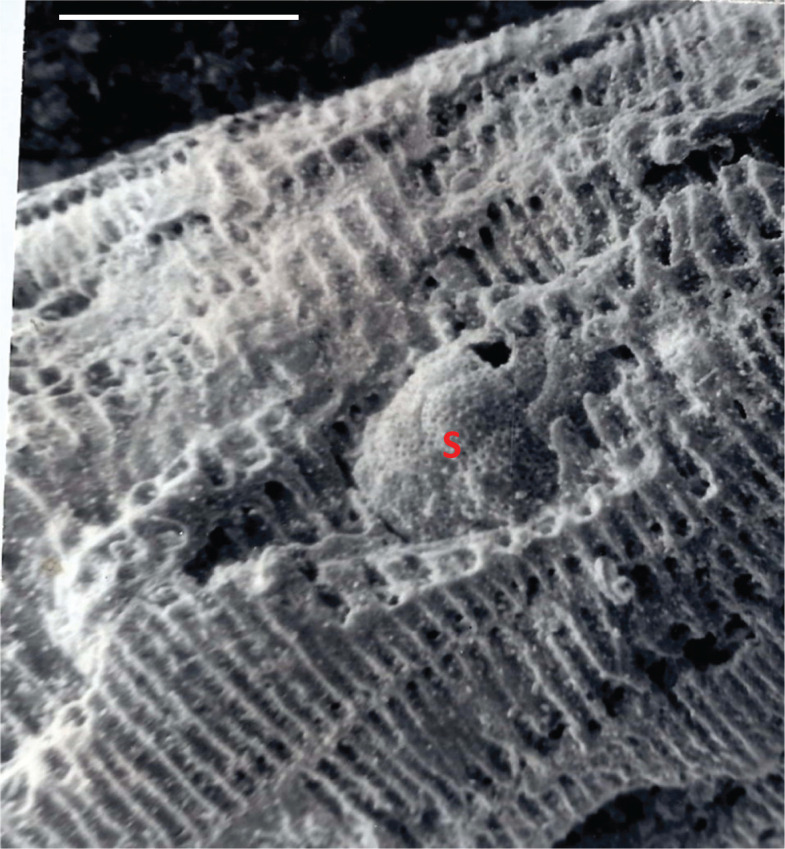
*Alveolinella quoyi* (d’Orbigny), Port Moresby, Coral Sea, New Guinea, solid specimen figured by BouDagher-Fadel [1], showing a parasite-symbiont(s) completely enclosed by the whorls, *Planorbulinopsis parasitica* Banner, embedded in the test. Scale bar = 0.25 mm.

The development of alveoles (blind recesses separated by septula; e.g. see Fig. 6) in some alveolinoids may have allowed them to harbour photosymbionts (e.g. diatom symbionts in living *Alveolinella* Douvillé [34], see Fig. 9). Endosymbiosis apparently played a major role in the evolution and diversification of the alveolinoids, and appears to be a key innovation that facilitated a change in habitat from an epifaunal, free-living mode of life, to one of living attached to phytal and non-phytal substrata.

On the other hand, the development of pillars in the fabulariids (see Fig. 7) and rhapydioninids may also be seen as providing mechanical strength to the test. For example, in discoidal forms, heavily pillared endoskeletons, as a rule, occur in forms living in very shallow, turbulent water. In the present day, robust and fusiform tests (as seen in *Alveolinella*) are adapted to a life in environments of moderate hydrodynamic energy, characteristic of water depths of less than 30 m, usually not in mobile sands, but rather on phytal or hard substrates such as reef rubble [1,35]. At Lizard Island, Australia, sand patches at ~15–20 m seemed to be the best habitat to collect *Alveolinella* (Hallock, personal comment). According to Hottinger [36], in *Alveolina* the function of the elongated fusiform test is related to motility, the test growing in the equatorial direction but moving in the polar direction. The living alveolinids (such as the elongate *Alveolinella*) appear to be confined to shallow-marine (down to depths of 80 m), clear, well-oxygenated, tropical and subtropical waters. They can hide or shelter under shallow layers of coral sand, in order to regulate the illumination that they require. Therefore, in comparing the occurrences of the extinct alveolinids with their Holocene descendants, we can safely deduce that these fusiform types, with large alveoles, thrived in sediments deposited in warm, shallow water. In fact, all alveolinoids are regarded as neritic (inner shelf) by Reichel [37], restricted to littoral, tropical, protected shelf and reef shoals [1,30,38], or moderate energy zones of the shallow ramp [39], and some genera are epiphytic [40].

## Phylogeny and palaeogeographic distribution of the Alveolinoidea

In this section, we will review the phylogenetic development and taxonomic relationships of the alveolinoids of the Cretaceous, Paleogene and Neogene. Furthermore, we clarify their provincial distributions, based around the Tethyan, American and Indo-Pacific provinces. We discuss the development of the three alveolinoid families, the Alveolinidae, the Fabulariidae and the Rhapydioninidae, which in the Cretaceous occurred in the Tethyan and American provinces, and then the later Cenozoic forms, of which the Alveolinidae and the Fabulariidae occurred in all three provinces (Tethys, American and the Indo-Pacific), while the Rhapydioninidae were constrained to the Americas.

### The Cretaceous Alveolinoidea

#### The Tethyan Province

The limestones of the Tethyan province range from what is today the Western Mediterranean, through the Middle East to the carbonate platforms of Tibet. This province saw the first development of the alveolinoids from small simple miliolids, such as *Pseudonummuloculina* (see Fig. 3). This latter is a simple small streptospiral-involute porcelaneous form, with an apertural slit or with a row of multiple apertures. It made its first appearance in the latest Hauterivian (PZ Hauterivian 2 [1,41]). It was widely distributed in the Tethyan province from the Albian until the early Paleogene [32,42–47], and is the ancestral form for many of the Alveolinoidea.

**Plate 1:**
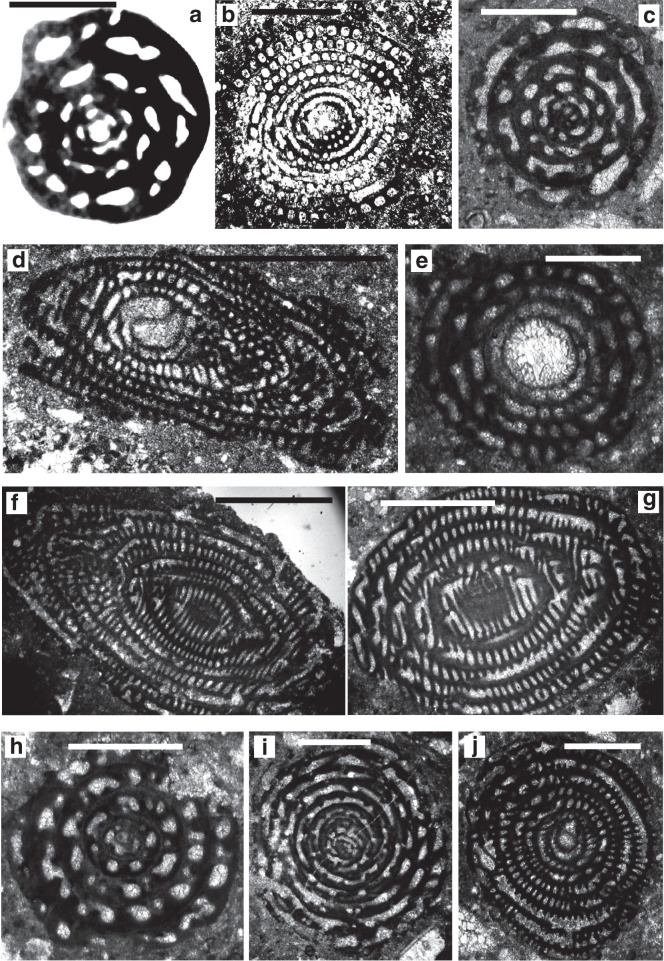
Scale bars: a, c, f, h = 0.5 mm; b, d, e, g, i, j = 1 mm. a. *Archaealveolina reicheli* (de Castro), figured by Loeblich and Tappan [48], Aptian, Italy. b. *Ovalveolina* sp., Cenomanian, France, UCL collection. c. *Ovalveolina crassa* Castro, Cenomanian, Iles Madam, UCL collection. d. *Praealveolina cretacea* Reichel, Cenomanian, Iles Madam, UCL collection. e–f. *Praealveolina tenuis* Reichel, Cenomanian, e) oblique equatorial section; f) oblique axial section, Audignon, near Saint-Sever, UCL collection. g–j. *Simplalveolina simplex* (Reichel), Cenomanian, Audignon, near Saint-Sever, UCL collection.

*Archaealveolina* (Plate 1, a) is the earliest defined **Alveolinidae**. It is subglobular with an early streptospiral stage, pre-septal passages and a single row of apertures at the base of the apertural face. This genus first appeared at the beginning of the Aptian and disappeared at the end of PZ Albian 1 [44]. *Archaealveolina* gave rise to and was replaced in the Albian by *Ovalveolina* (Plate 1, b–c), a globular form with planispiral coiling and large pre-septal passages [1]. *Ovalveolina* is recorded from many Tethyan localities ranging from PZ Albian 1 to PZ Cenomanian 3, but as discussed below, is not found in the American province until the beginning of PZ Cenomanian 2.

**Plate 2:**
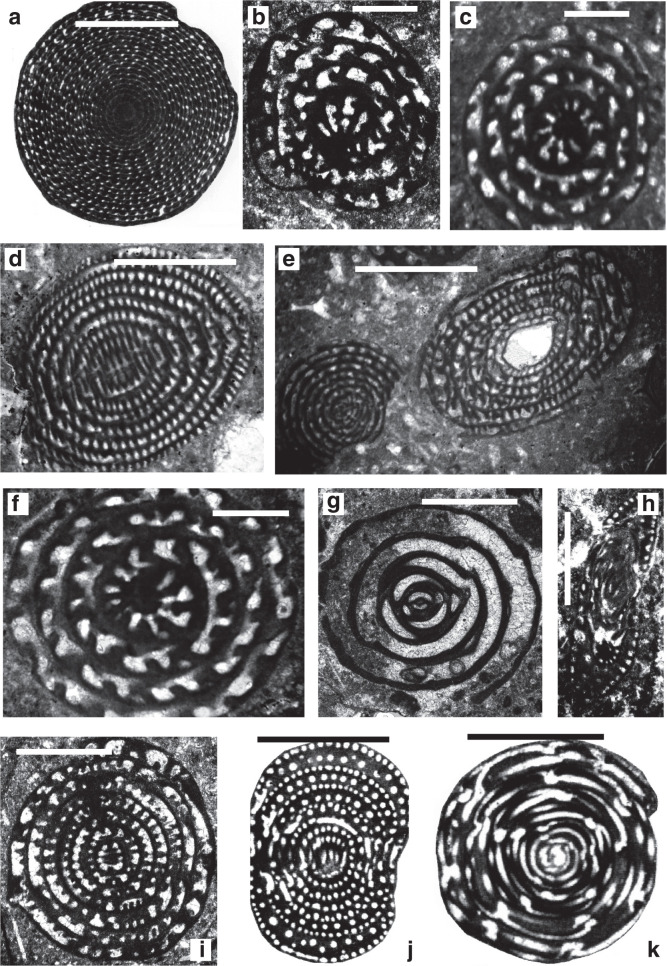
Scale bars: a = 0.15 mm; b–g, i, k = 0.5 mm; h, j = 1 mm. a. *Multispirina* sp., Cenomanian, Qatar, NHM M2425. b. *Streptalveolina* sp., Cenomanian, Iles Madam, UCL collection. c–f. *Cisalveolina lehneri* Reichel, Cenomanian, India, UCL coll. g. *Pseudonummuloculina* sp. Cenomanian, Paxos, Greece, P. Scholle coll. h. *Pseudedomia persica* Rahaghi, Cenomanian, Iran, Nanjing University coll. i. *Helenalveolina tappanae* Hottinger, Drobne and Caus, Cenomanian, Iles Madam, UCL collection. j–k. *Pseudochubbina globularis* (Smout) (= *Pseudedomia globularis* Smout), paratypes, Campanian, Iraq, NHM P42646.

In PZ Albian 4, the planispiral *Ovalveolina* gave rise to subglobular/fusiform *Praealveolina* (Plate 1, e–f) with much lower and narrower chamberlets. The apertural face of *Praealveolina* has, as does *Ovalveolina*, a single row of pores near the equator, however, the pores increase poleward to many rows of openings. *Simplalveolina* (Plate 1, g–j) which also appeared at PZ Cenomanian 1, is small and ovoid with numerous aligned septula, but lacks secondary chamberlets. The aperture is a single row of pores in the apertural face. In PZ Cenomanian 1, *Praealveolina* gave rise to the spherical *Multispirina* (Plate 2, a), with large, numerous chambers, interleaved by spires [37]. The aperture is formed by a row of pores in the apertural face of each of the multiple spires. The growth of *Multispirina* is an example of an alternative to elongate growth, where the multiple spires are a means of accelerating its growth [49]. The multiple spires also enhance the internal inter-connectedness of the pore spaces, as the numerous small apertures along the sutures connect the whorls in an irregular manner, thus making for short radial lines of interconnectedness throughout the test [33].

Parallel to the *Praealveolina* lineage, *Ovalveolina* also gave rise to the small to medium size globular, streptospirally coiled *Streptalveolina* (Plate 2, b; PZ Albian 4–Cenomanian 2). The latter also has large pre-septal passages and a single row of apertures on the apertural face. The trend in this lineage is to evolve planispiral later whorls, as in *Cisalveolina* (Plate 2, c–f; PZ Cenomanian 1–3), which has alternating septula with a simple narrow, slit-like aperture extending from pole to pole. *Cisalveolina*, unlike all the Cretaceous genera with pre-septal canals, has post-septal passages.

**Figure 10:**
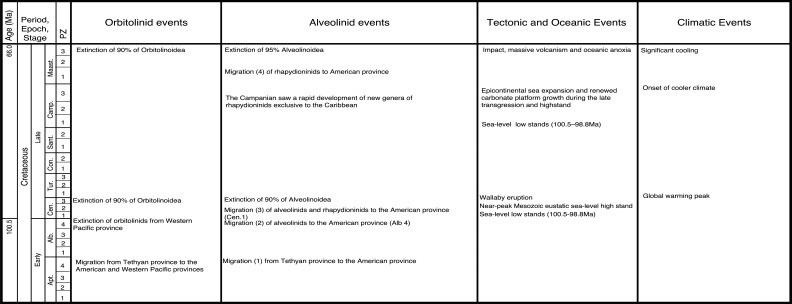
The major tectonic, oceanic and climatic events in the Cretaceous affecting alveolinoids and orbitolinid migration, evolution and extinction.

Of the early alveolinids, 85% did not survive the widespread oceanic anoxic event (OAE) at the Cenomanian–Turonian boundary (93.9 Ma), which may in turn have been triggered by the Madagascar and Caribbean volcanic provinces events (Fig. 10). The only survivor was *Praealveolina*, which in turn went extinct at the end of the Turonian.

The alveolinids re-appeared in the Late Cretaceous of the Tethyan province, but notably those that did appear had a streptospiral early stage and a planispiral-involute adult stage. It has been proposed that they are again phylogenetically linked to *Pseudonummuloculina* (Plate 2, g), and are an example of the homoplasic, re-appearance of morphologically identical form from a long ranging, robust small-form common ancestor [1,15]. They include the spherical *Helenalveolina* (Plate 2, i; PZ Coniacian 2–Santonian 2), *Subalveolina* (PZ Santonian 2–Maastrichian 3), and the axially elongated *Senalveolina* in the early Campanian. *Helenalveolina* is distinguished from the Fabulariidae by the absence of pillars subdividing the chambers, by its streptospiral coiling, and by the lack of a trematophore in its apertural face.

The earliest **Rhapydioninidae** originated in Tethys at the end of the Albian. The axially compressed rhapydioninids have homologous features to the alveolinids, and occupied similar niches, but developed separate parallel lineages. They evolved, as did the alveolinids, from the streptospiral *Pseudonummuloculina*, but did so later than the first appearance of the alveolinids [1,15,46,50]. They had a planispiral to streptospiral early stage, but diverged from the alveolinids lineages by becoming uncoiled, compressed, peneropliform-flaring, or cylindrical in later stages. The basal layer of the rhapydioninids is pierced by tubular supplementary passages as part of the ‘central thickening’ [51]. The rhapydioninid are comparable to modern peneroplid foraminifera, which commonly are found in shallow-water epifaunal habitats or on epiphytal hard substrates, including seagrasses and algal thalli [52–55].

**Plate 3:**
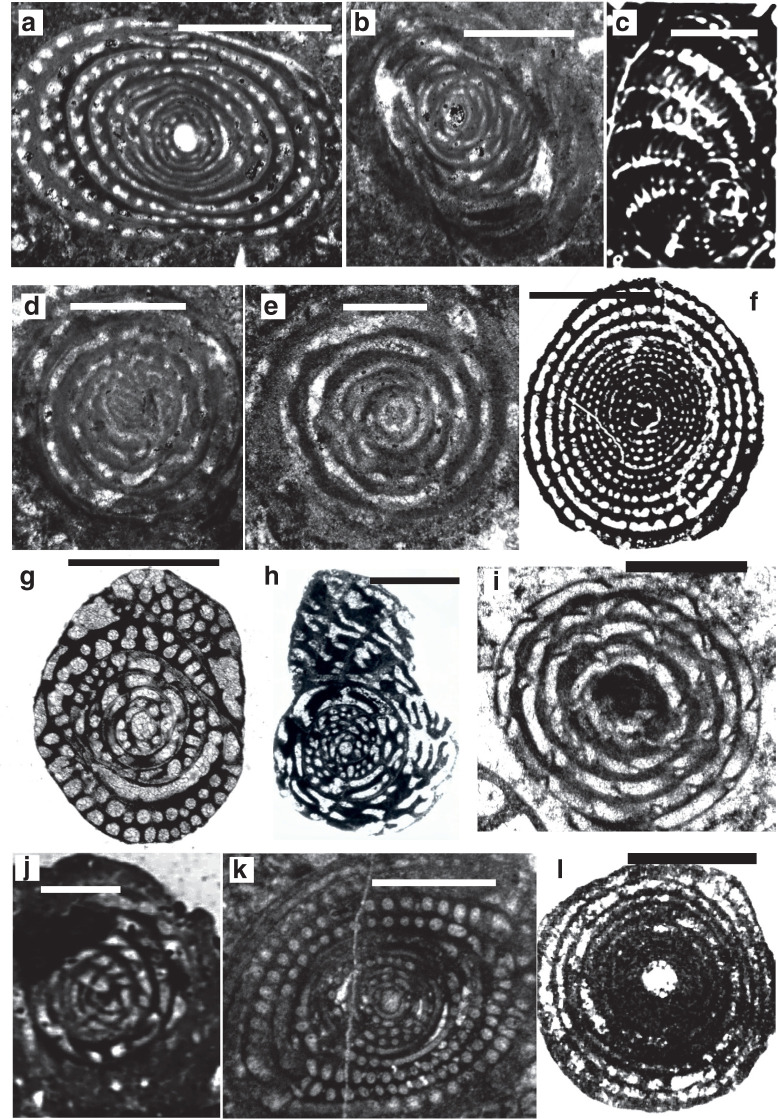
Scale bars: a–i, k–l = 0.5 mm; j = 1 mm. a–b. *Sellialveolina viallii* Colalongo, Cenomanian, Xinyu, Nanjing University coll. c. *Murciella cuvillieri* Fourcade, figured by Fourcade [63], Campanian, Spain. d–e. *Periloculina iranica* Rahaghi, Cenomanian, Iran, Nanjing University coll. f. *Lacazina* sp., Coniacian, India, UCL coll. g–h. *Chubbina cardenasensis* (Barker and Grimsdale), g) Late Cretaceous, Cardenas, San Luis Potosi, Mexico, NHM P33044; h) figured by Robinson [10] as a co-type megalospheric specimen of *Chubbina jamaicensis* Robinson, Campanian, Jamaica, NHM P48053. i. *Glomalveolina pilula* Hottinger, Ypresian, Lower Laki Formation, Pakistan, UCL coll. j. *Glomalveolina* sp., Eocene, Libya, UCL coll. k. *Glomalveolina lepidula* (Schwager), Early Eocene, Longjiang section, Tibet, Nanjing University coll. l. *Pseudofabularia matleyi* (Vaughan), figured by Loeblich and Tappan [48], Middle Eocene, Chapelton Formation, Jamaica.

*Sellialveolina* (Plate 3, a–b), was the most primitive rhapydioninid and occurred across Tethys between PZ Albian 4–Cenomanian 4 [17,56]), colonising the carbonate platforms from the Middle East to the Iberian Peninsula. Unusually, it is a cosmopolitan rhapydioninid, and is reported from the American province from PZ Cenomanian 2–Turonian 2 (n.b. in the Maastrichtian, *Rhapydionina* becomes the only other example of a cosmopolitan rhapydioninid). *Sellialveolina* has a lenticular, planispirally-enrolled test, where the chamber lumen is subdivided into numerous chamberlets by septula. *Sellialveolina* species increased in overall test size and complexity of internal structures during their stratigraphic evolution.

Tethyan rhapydioninids are provincially characterised by *Pseudedomia* (Plate 2, h) and its descendants, which evolved from *Sellialveolina* in PZ Cenomanian 1. They are common in the Late Cretaceous deposits of the European–North African region [51,57–62]. This lineage exhibits an evolutionary trend with an increasing diameter of the megalospheric form, a reduction in the streptospiral nepionic stage, and an increase in the differentiation and regularity of the peripheral chamberlets in comparison with the median chamberlets in the same chamber [50].

*Sellialveolina* and *Pseudedomia* both resemble *Ovalveolina* in their globular initial part, but develop compressed, curved chambers in their final whorls. The chambers in *Pseudedomia* become progressively longer, strongly overlapping the preceding chambers. The evolutionary lineage from *Pseudedomia* showed progressive uncoiling and cylindrical development through *Rhapydionina, Murciella* (Plate 3, c) and *Cyclopseudedomia* (see Fig. 7). The ‘central thickening’ of *Rhapydionina* compares with the thickened basal layer in the polar area of *Subalveolina* [64]. *Murciella* has crosswise, oblique, median tubular chamberlets (the ‘helicoidal’ structures of Fleury [65]), while *Cyclopseudedomia* is cyclical with compressed arched chambers in its final whorls.

We believe that the Tethyan *Murciella* has sometimes been misidentified as a *Raadshoovenia*, which in fact is an American Paleocene form (see below). For example, the Late Cretaceous Tethyan *Raadshoovenia* [e.g. *R. salentina* (Papetti and Tedeschi)] has the same advanced early planispiral-involute nepiont as *Murciella*, and the two genera were considered synonyms by De Castro [57]. We agree with this analysis and contend that all Cretaceous, Tethyan *Raadshoovenia* are in fact synonyms of *Murciella.*

All Tethyan rhapydioninids, including *Murciella*, went extinct at the end of the Maastrichtian. The true *Raadshoovenia* appeared in the Paleogene of the American province. There are no records of *Raadshoovenia* from the Paleocene of the Tethyan province. *Raadshoovenia* has a streptospiral nepiont in both megalospheric and microspheric generations, and characteristically has a smaller test than *Murciella*. *Murciella* and *Raadshoovenia* evolved in different times on the two sides of the Atlantic. They are again an example of homoplasic evolution, rather than representing a single genus that survived the events at the Cretaceous–Paleogene boundary while simultaneously moving provinces.

The **Fabulariidae** are also common in similar ecological niches as the alveolinids, but they did not appear before the Late Cretaceous and were endemic to the Tethyan province, with no record from the Cretaceous deposits of the American province. They arose directly and independently from the alveolinids and the rhapydioninids, either from the simple *Quinqueloculina* group [66], or they had a true miliolid origin from, for example, the simple-form *Idalina* (see [1]). The fabulariid test is large, dimorphic, multi-chambered with miliolid coiling, tending to become reduced in subsequent growth stages, either to bilocular or to monolocular chamber cycles, with a trematophore as the aperture. According to Hottinger ([67], p. 4) ‘A trematophore, or a sieve constituting the face of many porcelaneous larger foraminifera, is in miliolids produced by the coalescence of teeth, covering a large pre-septal space. It may be supported by residual pillars’. Chambers have a thickened basal layer, subdivided by pillars or secondary partitions. Foraminifera in the adult growth stages have a fixed apertural axis. For the terminology and the orientation of sections needed for detailed structural analysis see Drobne [68] and Hottinger et al. [15].

*Idalina*, a simple miliolid with a quinqueloculine to biloculine test, gave rise to *Periloculina* (Plate 3, d–e; see [15]), which has incomplete partitions and an aperture consisting of a round trematophore with a bar-like tooth. Also, in the Coniacian, *Lacazina* (Plate 3, f) appeared, with chambers that overlap throughout, with a concentric growth pattern, and with an annular trematophore (Figs 3 and 7) [1,69]. *Lacazina* is reported from many places in the Tethyan province, for example, from Southern Europe [32,38], France [48,60,70], Spain [70–72], Sardinia [15,60], and Israel [48,71,72]. In *Pseudolacazina* the chambers are subdivided by longitudinal partitions or by pillars supporting the chamber roof, while in *Pseudochubbina* (Plate 2, j–k) parallel anastomising passages are present in the basal layer. Correcting BouDagher-Fadel [1], we now believe that all Cretaceous fabulariids, except the small, primitive, robust *Idalina*, went extinct at the K–P boundary.

#### The American Province

**Figure 11:**
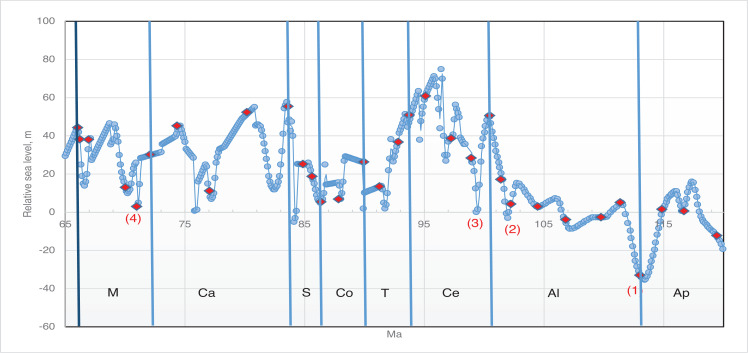
Variation in sea level during the mid to Late Cretaceous based on Miller et al. [73]. The red diamonds represent the planktonic foraminiferal zones, while the numbers signify the main migration events of the alveolinids.

The mid-Cretaceous **Alveolinidae** were extremely rare in the American province, and post-date the Tethyan forms. It is inferred that they migrated from the Tethyan province after their first appearance there. The pre-existing Tethyan *Pseudonummuloculina* appeared in the Americas together with some Tethyan orbitolinds (see [2]; Fig. 3) during the sea-level low stand of the Aptian–Albian boundary (migration 1 in Fig. 11), and is represented in both provinces by *P. heimi* (Bonet). This species is widely distributed in the Albian–Cenomanian of both Western Tethys and the Gulf Coast [74–76]. It has also been recorded from Texas, Florida and Louisiana in the United States [77].

The streptospiral alveolinids appeared in the American province, in PZ Albian 4b, and are represented by *Streptalveolina mexicana* Fourcade, Tardy and Vila [78]. This species initially described from the early Cenomanian, Mexican Aurora Formation, has also been recorded in some localities from PZ Albian 4 to Cenomanian 2 of the Gulf Coast [79,80]. It is endemic to the American province, but appears to be virtually identical to the PZ Albian 4a, Mediterranean species *S. peybernesi* [81], and seems to have appeared in America after the late Albian sea-level low stand (migration 2 in Fig. 11). Finally, the pre-existing Tethyan *Ovalveolina maccagnoae* De Castro is reported from the Americas from PZ Cenomanian 2 [82] from the mid-late Cenomanian deposits of the Manantlan Range in Colima State, Mexico, which again correlates with a global sea-level low stand (migration 3 in Fig. 11).

In the American province, the **Rhapydioninidae** are of two types, represented by the mid-Cretaceous *Sellialveolina*, and the distinct, later Cretaceous *Chubbina*, *Chubbinella, Praechubbina, Caribalveolina* lineage.

*Sellialveolina* is the most primitive genus of the axially compressed Alveolinoidea, and is the only genus of the Rhapydioninidae that is found in the mid-Cretaceous of the American province. It first appeared as a Tethyan form in PZ Albian 4, but it is reported in the Americas 3.4 Ma later (in PZ Cenomanian 2) as *Sellialveolina* sp. and *Sellialveolina* cf. *drorimensis* (a Tethyan form) from northern Peru [83]. This appearance in the Americans coincides with that of *Ovalveolina*, which as noted above correlates with a global PZ Cenomanian 2, sea-level low stand (migration 3 in Fig. 11).

The later American rhapydioninids forms have no Tethyan equivalents, and seem to have evolved directly from the American *Pseudonummuloculina.* They are described from several localities in southern Mexico, Guatemala and in the Caribbean [10,18,84–86].

They appear in PZ Campanian 1, and rapidly develop the *Chubbina* (Plate 3, g–h) lineage, which is endemic to the American province. The most primitive genus of this lineage, *Praechubbina*, with a poorly developed endoskeleton, formed by short, incomplete septula and floors, is characterised by a single aperture in its early stages. It differs from the ancestor genus *Pseudonummuloculina* by the presence of endoskeletal elements and multiple apertures in adult stages of growth [18,84]. The septula in *Chubbinella* and *Chubbina* are complete, and divide the chambers into long tubular chamberlets. The septula of *Chubbinella* form chamberlets of equal calibre within the same chamber, while in *Chubbina* the chamberlets are unequal and the median chamberlets are parallel to the peripheral chamberlets. In *Chubbina* the subdivision of the chambers begins in the early stages of growth, whereas in *Chubbinella* the early parts are undivided [17]. On the other hand, the axially elongated *Caribalveolina* has alveoles and chubbinid features in the poles that are similar but less regular than the polar features of *Praealveolina* [50].

**Figure 12:**
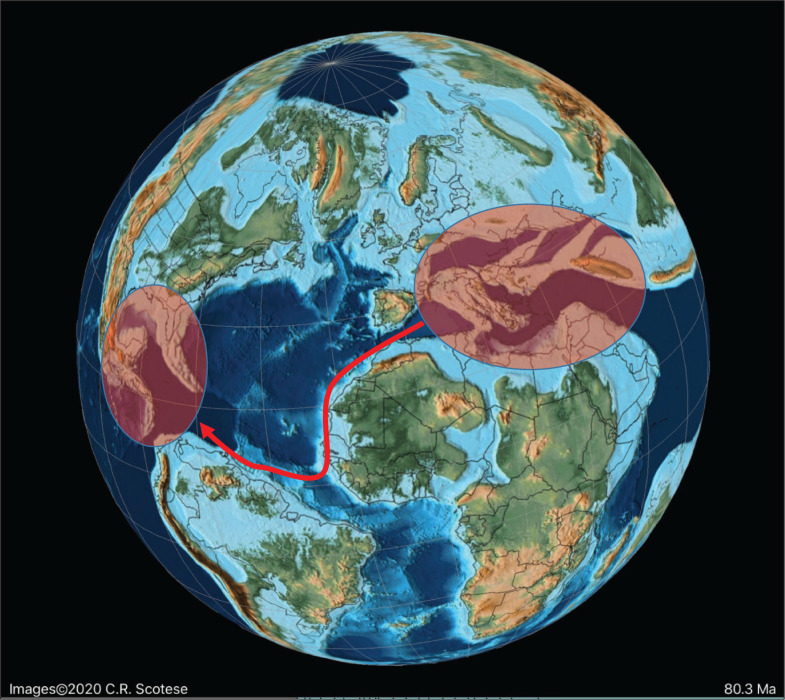
Schematic migration of alveolinoids during Late Cretaceous, shown by red arrows, from the Tethyan province to the American province.

A Late Cretaceous migration of the rhapydioninids from the American province to Tethys never occurred. However, towards the middle Maastrichtian (PZ Maastrichtian 2), migration of the Tethyan form *Rhapydionina* to the American province did occur, with *Rhapydionina cf. R. liburnica* (Stache) reported from PZ Maastrichtian 4 of south east Mexico [86]. The early Maastrichtian low sea-level stands (migration 4 in Fig. 11) correlate with the timing of this apparent trans-oceanic migration (Fig. 12).

All American rhapydioninids went extinct at the K–P boundary.

### The Paleogene Alveolinoidea

No complex Alveolinoidea survived the Cretaceous/Palaeogene (K–P) extinction, however their ancestral small and simple miliolids were robust enough to continue (Fig. 8). In the Paleogene, these ancestral forms again gave rise to new members of all three of the Alveolinoidea families, which closely echoed their Cretaceous predecessors. The alveolinids of the Paleogene show a complex history of regeneration from the simple *Pseudonummuloculina* (which survived the end Cretaceous event and only went extinct in the late Thanetian, P4), with new forms occurring independently in both the Americas and Tethys. In the Eocene, migration of alveolinids again occurred, but as previously seen in our studies of the rotaliids, now in a west to east direction, with migration of forms from Tethys to the Indo-Pacific.

**Figure 13:**
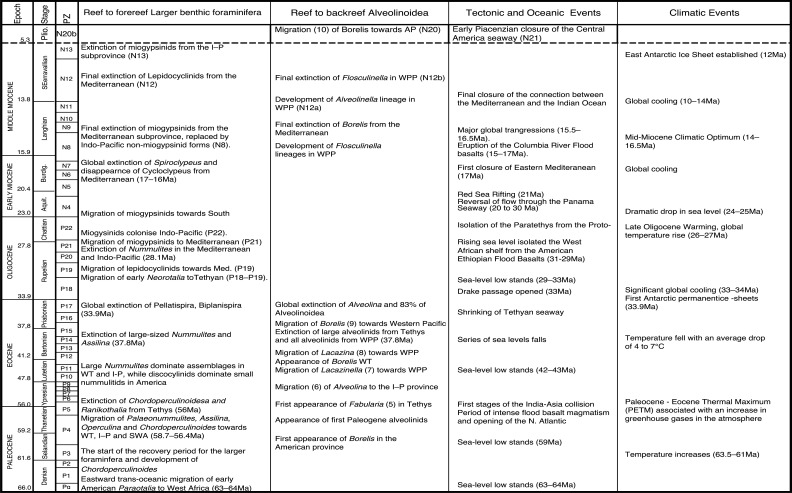
The major tectonic, oceanic and climatic events in the Neogene affecting alveolinoids migration, evolution and extinction. WPP, I-P = Indo-Pacific; WT = Western Tethys; A-P = American province.

**Figure 14:**
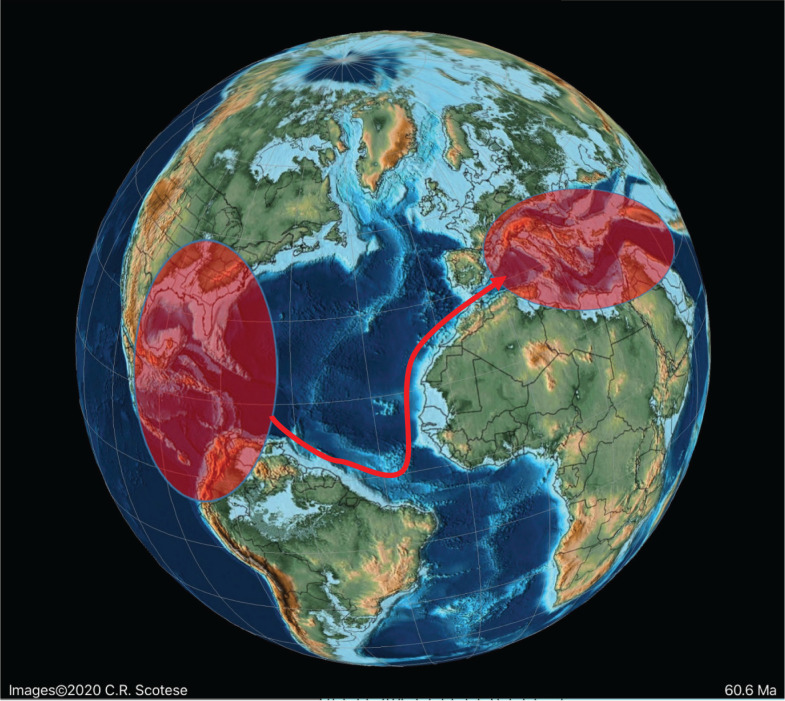
Schematic migration of alveolinoids during Early Paleogene, shown by red arrows, from the American province to the Tethyan province.

**Figure 15:**
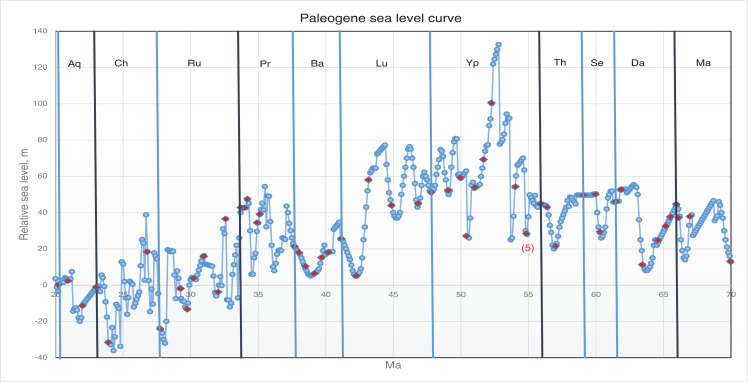
Variation in sea level during the Paleogene based on Miller et al. [73]. The red diamonds represent the planktonic foraminiferal zones, while the numbers signify the main migration events of the alveolinids.

**Figure 16:**
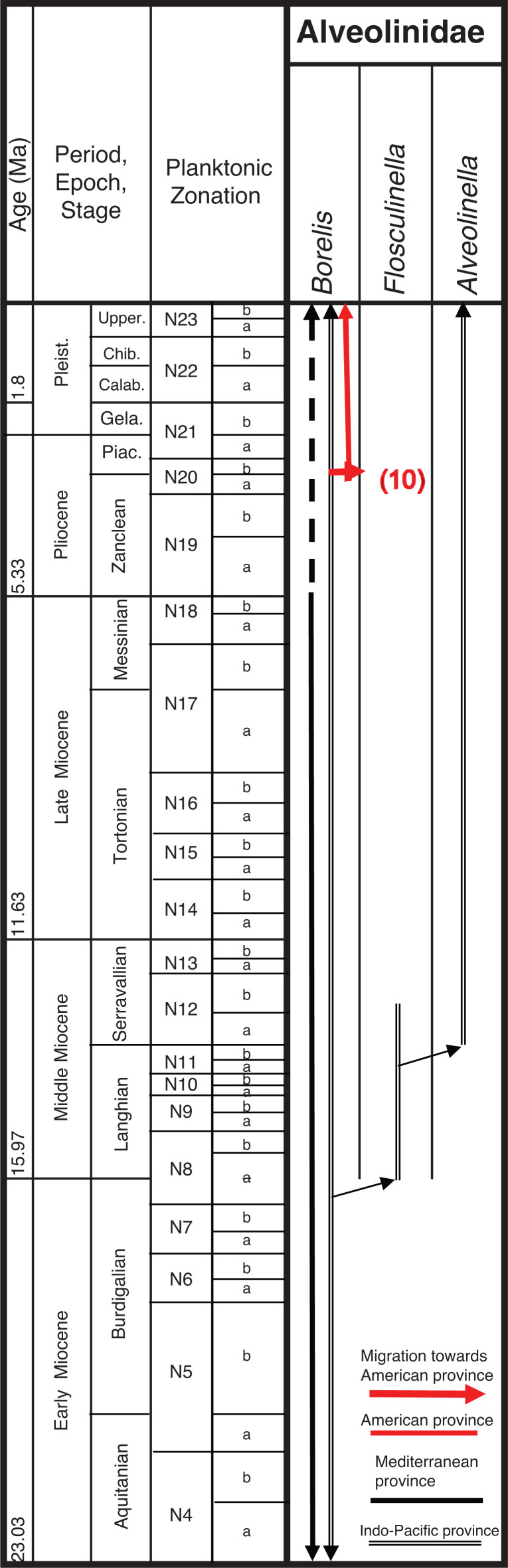
Ranges of the main genera of the Neogene Alveolinoidea in the three provinces.

In the Paleocene, the rhapydioninids also re-appeared from a small miliolid after the K–P extinction, but are only found in the Americas (Fig. 8). In Tethys the fabulariid ancestral form *Idalina* survived the Maastrichtian extinction, and gave rise to a second wave of Paleocene forms. They gave rise in the Middle Paleocene to a *Periloculina* lineage, which echoes the homomorphic Late Cretaceous lineage, but which is separated by a stratigraphic interval of over 6 Ma. In yet another example of homoplasy, *Fabularia* evolved in the Americas independently of the other Tethyan fabulariids, and migrated during an early Ypresian sea-level low stand to Tethys (Figs 13–15). All rhapydioninids and fabulariids went extinct before the end of the Paleogene, and only an alveolinid, *Borelis*, survived into the Neogene (Fig. 16).

#### The American Province

The **alveolinids**, except for a couple of primitive genera (not discussed in [1]), are rare in the Paleogene of the American province. A primitive small globular planispirally, coiled form, *Quasiborelis* Hanzawa [e.g. *Q. gunteri* (Cole)] with streptospiral early chambers, was reported by Hanzawa [87] from Florida (Fig. 8). It has a simple internal structure, consisting of a poorly developed endoskeleton divided by few septula and floors, and high pre-septal canals, and it must have evolved from a small miliolid that survived the K–P event. *Quasiborelis* was short ranged, but was followed directly by forms similar to the later Tethyan *Borelis* (see [19]). The American *Borelis* is subglobular in shape with only one row of chamberlets and a planispiral later stage, and has been reported from the Middle Paleocene to the Early Eocene (PZ P3–P9) of the American province [18,19,88–90]. The American *Borelis* occupies similar niches as the Tethyan *Glomalveolina* (Plate 3, i–j; [19]), which is abundant in the very low hydrodynamic energy back-reef environments of Tethys, but is absent from the American province. The American *Borelis* has aligned septula and pre-septal passages, but they are generally smaller in size than those of the Tethyan *Borelis*. American *Borelis* and Tethyan *Borelis* are probably examples of homoplasy, as they do not co-exist, and are in fact separated by 6.6 Ma in the geological column.

The **Rhapydioninidae** were common and mainly endemic to the Late Cretaceous low-latitude shallow-water reefal and lagoonal settings of the American province. All of them, however, went extinct at the end of the Cretaceous. The rhapydioninids of the Paleogene are also distinct from those described from the Cretaceous of the Tethyan province, and are only found in the American province. The genus *Raadshoovenia (R. guatemalensis* van den Bold) was originally established by van den Bold [91] from the Eocene of Guatemala, however, as discussed above, it is different from the *Raadshoovenia* misidentified in the Tethys, which are in fact *Murciella*. As is also discussed above, the appearance of *Raadshoovenia* in the Paleocene–Eocene of the American province is therefore yet another example of homoplasy, where similar characters arose by convergent evolution. True *Raadshoovenia* has been reported from Mexico [92,93] and Guatemala [18,51,57]. There are no Paleocene records of *Raadshoovenia* from the Tethyan province.

The American *Raadshoovenia* gave rise to *Neomurciella*, which made its first appearance in the Selandian of the American province (PZ P3a [18,94]). *Neomurciella* show a crosswise development of the central and marginal chamberlets, as was found in the Campanian–Maastrichtian *Murciella* gr. *M. renzi* Fleury (see [18,65,50]), but this reappearance is 4.4 Ma later than the disappearance of *Murciella*. *Neomurciella* survived the Paleocene–Eocene boundary only to disappear within the Early Eocene.

The American **Fabulariidae** are represented by members of the genus *Fabularia*. This genus evolved for the first time in the American province from the small, primitive form *Miliola* in the Early Eocene (Ypresian, PZ P5b) by developing an involute, biloculine last stage after the early quinqueloculine stage, and thickened vertical partitions, which subdivide the chambers into elongated chamberlets with two tiers in the outer whorls. The two chambers per whorl is a key characteristic of the megalospheric forms (see [1]). The fabulariids were represented in the American province by endemic species, such as *F. vaughani* Cole, *F. cassis*, *F. colei* and *F. verseyi*. These species are only found in the Caribbean region [32,95,96] from the Early to Middle Eocene (PZ P6–P12a). In the Middle Eocene, Lutetian (PZ P10), the biloculine *Fabularia* (Fig. 7) gave rise to *Pseudofabularia* (Plate 3, l) by developing alveolinid-like pre-septal passages [97]. The Bartonian–Priabonian fabulariid assemblages manifested gigantism, however, they all disappear at the end of the Eocene, except for *Fabularia verseyi*, which survived the Eocene–Oligocene extinction, only to die out in the Rupelian [98,99].

#### The Tethyan Province

Although the Tethyan province was still the hotspot for **Alveolinidae** diversity, the Paleocene forms evolved independently from their Late Cretaceous relatives (Fig. 8). In the Tethyan province, they re-appeared during the warm Late Paleocene (Thanetian), where they colonised reefal and backreef environments. During the Eocene, they became large and abundant in forereef environments, only to largely disappear by the end of the Eocene.

Paleogene alveolinids presumably evolved, as did the Cretaceous forms, from *Pseudonummuloculina* (see [15,67]). They subsequently develop lineages that are spherical to fusiform in shape, and planispiral in the adult part, exhibiting morphological convergence (homoplasy) with the fusiform/globular alveolinids of the mid-Cretaceous (e.g. the *Praealveolina* group). A considerable gap, however, of about 20 Ma exists in the fossil record between the Alveolinidae of the Paleocene and the mid-Cretaceous *Praealveolina* group.

**Plate 4:**
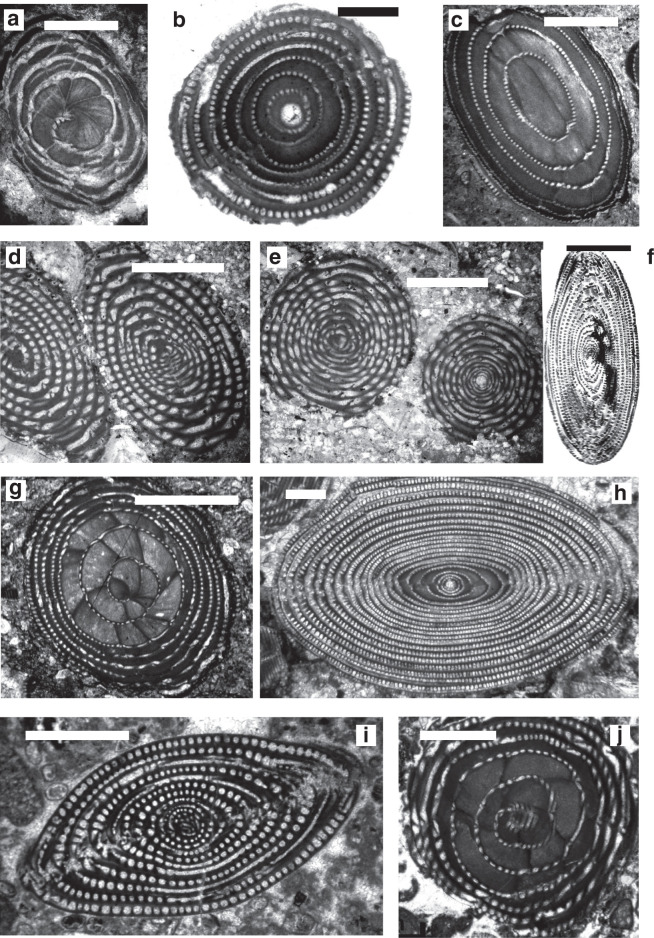
Scale bars: a–j = 1 mm. a–c. *Alveolina globosa* (Leymerie), Ypresian, Eocene, Laki Limestone (Laki Formation), Sakesar Peak in the Salt Range, Pakistan, a) UCL coll.; b) NHM Davies coll.; c) Toraja Formation (Sulawesi), SEA coll., Royal Holloway. d–f. *Alveolina oblonga* d’Orbigny, Early Eocene, d–e) Dunghan, Siah Koh, UCL coll; f) Chaussy, Val-d’Oise, France, UCL coll. g. *Alveolina leupoldi* Hottinger, Early Eocene, Qumiba section, Tingri, Tibet, Nanjing University. h. *Alveolina elliptica* (Sowerby), Eocene, Afghanistan, NHM Skinner coll. P7404. i. *Alveolina corbarica* Hottinger, Early Eocene, Longjiang section, Tibet, Nanjing. j. *Alveolina daniensis* Drobne, Early Eocene, Ladakh region, India. Sagar Damania, Indian Institute of Technology coll.

**Plate 5:**
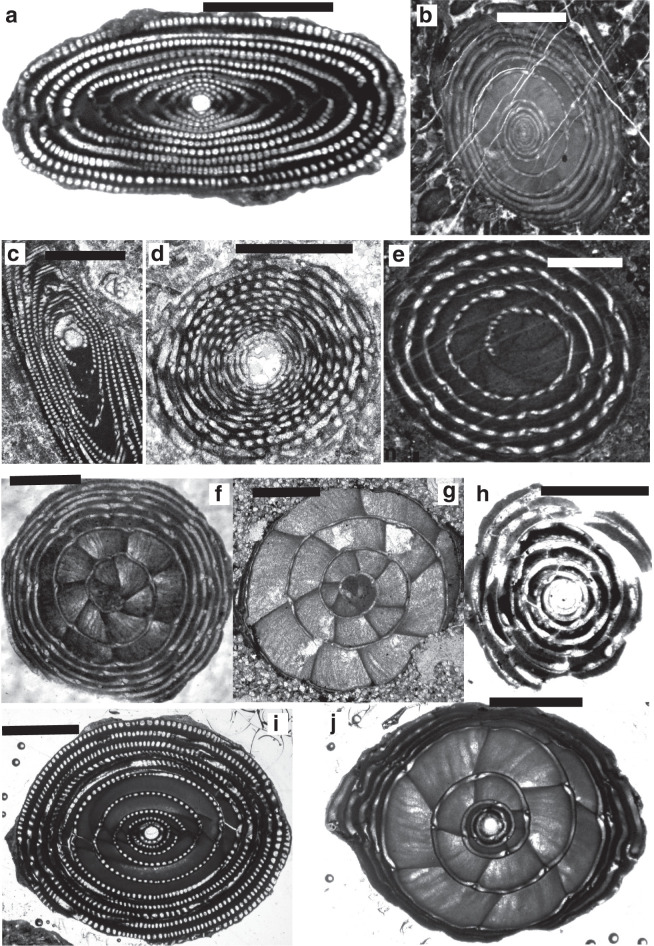
Scale bars: a–j = 1 mm. a. *Alveolina vredenburgi* Davies 1937 (= *Alveolina cucumiformis* Hottinger), topotype, axial section, Late Paleocene, Aquitaine, France, UCL coll. b. *Alveolina pasticillata* Schwager, Early Eocene, Ladakh region, India, Sagar Damania, Indian Institute of Technology coll. c–d. *Alveolina stipes* Hottinger, Middle Eocene, Ainsa, Spain, UCL coll. e. *Alveolina solida* Hottinger, Early Eocene, Ladakh region, India, Sagar Damania, Indian Institute of Technology coll. f. *Alveolina leupoldi*, Hottinger, Early Eocene, Coustouge, France, UCL coll. g. *Alveolina palermitana* Hottinger, Middle Eocene, Middle Khirthar, Pakistan, UCL coll. h. *Alveolina vredenburgi* Davies, topotype, equatorial section, Late Paleocene, Aquitaine, France, UCL coll. i–j. *Alveolina subpyrenaica* Leymerie, Early Eocene, Zagros, Iran, UCL coll.

**Plate 6:**
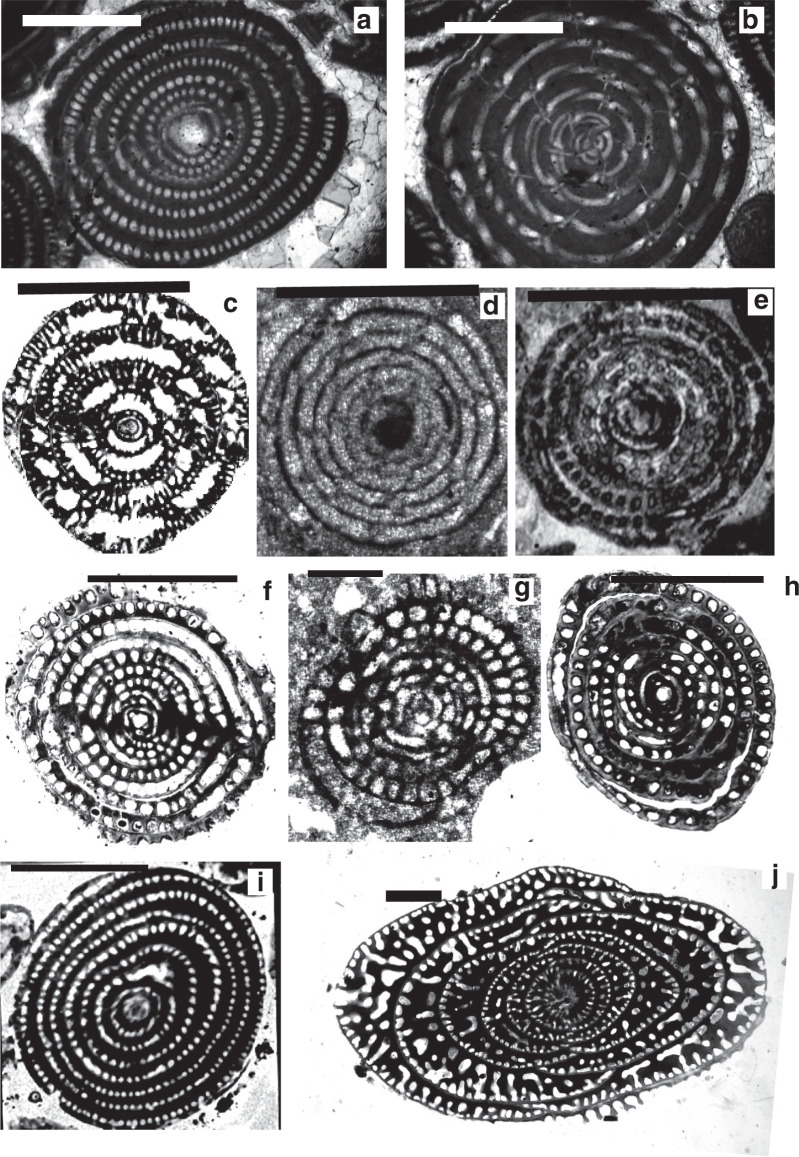
Scale bars: a–j = 1 mm. a–b. *Alveolina laxa* Fottinger, Early Eocene, Egypt, UCL coll. c. *Globoreticulina iranica* Rahaghi, figured by Hottinger [67], Middle Eocene, Shiraz, Iran. d. *Borelis pygmaeus* Hanzawa, Oligocene, Borneo, 65/9 Loc.205, UCL coll. e. *Borelis vonderschmitti* (Schweighauser), Late Eocene, Syria, UCL coll. f. *Borelis curdica* (Reichel), Miocene, Turkey, NHM coll. g. *Borelis melo* (Fichtel and Moll), Middle Miocene, Turkey, NHM P49087. h. *Borelis haueri* (d’Orbigny), Middle Miocene, Baden-Brickyard, Baden, Australia, UCL coll. i. *Lacazinella wichmanni* (Schlumberger), Late Eocene, Indonesia, UCL coll. j. *Fabularia discolithus* Defrance, Eocene, calcaire grosier de Rennes, France, NHM Brady coll. P41603.

The evolutionary lineages of the Paleogene alveolinids show an incremental increase in the length of the test from pole to pole, from globular through ovoid to elongate, and spindle-shaped. The oldest Paleogene form, *Glomalveolina* (Plate 3, i–k), with a streptoloculine origin and with a fixed axial coiling throughout its ontogeny, is considered the ancestor of *Alveolina*. *Glomalveolina* is small, globular and non-flosculinised, with a spherical proloculus in a streptospiral coil and a later planispiral stage. The streptospiral coil still exists in the microspheric forms of *Alveolina. Alveolina* (Plate 4, a–j; Plate 5, a–j; Plate 6, a) is planispiral–fusiform with a single tier of chamberlets in each chamber and, unlike *Glomalveolina*, shows considerable thickening of the basal layer in the equatorial zone (flosculinisation). Pre-septal and post-septal passages are present in both *Glomalveolina* and *Alveolina.*

In the Eocene the amount of flosculinisation varies in different species of *Alveolina*, and might be considered to be of specific value only. *Alveolina* evolve into larger forms through the Eocene, and more than 90 species of the genus *Alveolina* have been reported in the Mediterranean [8]. Many of the species reached gigantic sizes (up to 20 mm long [100]) before the end of the Middle Eocene, where they become essential members of Eocene carbonate fossil assemblages. *Alveolina* species, however, became smaller again before completely disappearing at the end of Eocene (see [1]).

In the Eocene *Alveolina* gave rise to new genera, such as *Globoreticulina* (Plate 6, c) and *Malatyna*, which exhibited similar trends of evolution to the Cretaceous *Subalveolina* and *Praebullalveolina.* They developed streptospiral nepionts in both generations and a planispiral-involute chamber arrangement in the adult stage of growth [67,101]. Both these genera became extinct at the end of the Lutetian.

Paleoclimatic evidence shows a trend of cooling during late Middle Eocene and at the Eocene–Oligocene (E–O) boundary ([102]; Fig. 13). At the Middle to Late Eocene boundary (37.8 Ma) major changes in global climate and ocean circulation [102] were likely responsible for the disappearance of the gigantic alveolinid species seen at the end of the Bartonian (see [1]). The E–O boundary cooling period followed the onset of the Late Eocene rapid sea-level changes, which led to the disappearance of vast carbonate platform and lagoonal environments, and the final extinction of *Alveolina*, and indeed of 85% of all Alveolinoidea. The move into an ‘ice house’ climate may have been triggered at this time by the opening of the Tasmanian gateway [103].

The only alveolinid survivors of the E–O cooling event were forms related to *Borelis* (Plate 6, d–h), which is in fact an extant alveolinid, and the only one to survive into the Neogene. The appearance of *Borelis* in the Tethyan province in the Bartonian was 6.6 Ma years after the last recorded *Borelis* species in the Paleogene of the American province. Given the magnitude of this gap in the stratigraphic column, we suggest that it is unlikely that this appearance in Tethys was the result of a migration event. We suggest that it is more probable that this appearance in Tethys is yet another example of homoplasy, where re-evolution from a primitive form gave rise to a form in Tethys that was generically similar to the extinct American form, and that it filled the Tethyan niche previously occupied by *Glomalveolina.*

In the Tethyan Eocene, *Borelis* is represented by the spheroidal *B. vonderschmitti* (Schweighauser), which was widespread throughout the Bartonian and Priabonian of Western Tethys [104,105]. *Borelis* is common in deposits laid down in Early Oligocene, low-latitude, shallow-marine environments, and differs from *Alveolina* in having only a pre-septal passage and a secondary small tier of chamberlets, which alternate with the larger ones producing ‘Y’ shaped septa in the axial section [106]. In the Oligocene, the sub-spheroidal, small (0.5–1.5 mm in diameter and 0.4–1.2 mm long), tightly coiled *Borelis inflata* (a form with no Y-shaped septula, see [105]) is also recorded from the Oligocene to the Tortonian of the Western Tethys [107–110].

In the Early Oligocene (from about 33.9 Ma), the Drake Passage opened and there was further climatic cooling and ice volume increase ([102]; see Fig. 13). *Borelis* species, which survived the E–O boundary, became adapted to globally cooler environments, while others migrated to the warmer Tethys, such as *B. inflata.*

The Oligocene continue to witness an evolutionary trend of small alveolinids in the Mediterranean, such as *Bullalveolina.* This form evolved from being streptospiral to having a planispiral quinqueloculine test, but unlike the extant *Borelis, Bullalveolina* disappeared at the end of the Oligocene. Generally, however, there were very few notable extinctions of LBF at the end of the Oligocene (see [1]). This stratigraphic boundary is probably connected to plate tectonic events, such the development of the Alpine–Himalayan orogeny, which were more gradual events and did not seem to trigger major extinctions, or to gradual changes in climate caused by the growing thermal isolation of Antarctica as Australia drifted northwards [102].

As in the Cretaceous, the Paleogene **Fabulariidae** (mainly the lacazinoform genera, *Lacazina*, *Lacazinella*) are directly derived from the small primitive *Idalina* which survived across the K–P boundary (see Fig. 8). Further to the ranges listed in BouDagher-Fadel [1], it now seems that the Fabulariidae re-appeared, independently from their Cretaceous counterparts. In doing so, they developed morphologically convergent, homoplasic lineages, which has led to these lineages being given the same generic names as their Cretaceous counterparts. However, we again contend that the considerable stratigraphic gap between their reappearance in the Middle–Late Paleocene and the end Cretaceous forms excludes any chance of a direct phylogenetic relationship, other than via a homoplasic repeated evolution from the same *Idalina* common ancestor. There are notable differences between Cretaceous and Paleogene genera, so for example, all Paleogene representatives of *Pseudolacazina* are distinguished from the Cretaceous forms in having lower in shape chambers, subdivided by continuous chamber partitions (septula) (see [15,111]).

In the Paleocene, *Periloculina* and *Pseudolacazina* echoed the Cretaceous forms, but they uniquely gave rise to Paleogene *Lacazina* and *Lacazinella* by gradual ontogenetic transformation from pluri- to unilocular, completely overlapping growth. The two chambers per whorl is a key characteristic of the megalospheric forms in the fabulariid, however, in *Lacazina* and *Lacazinella*, the microspheric tests have a small initial biloculine stage, followed by uniloculine growth, while their megalospheric tests only show uniloculine development, with a large proloculus. The basal layer of *Pseudolacazina* bears radial pillars supporting the chamber roof that evolve into low longitudinal septula, which do not reach the chamber floor in *Lacazinella*, to continuous septula supporting the external chamber wall in *Lacazina* [1,112].

The re-appeared *Lacazina* is first recorded in Tethys from the Selandian (PZ P4a), while *Lacazinella*, having completely overlapping chambers, is rare in the Tethyan province and has not been found in Europe, but is only recorded from Oman and other parts of the Middle East [113].

On the other hand, the American *Fabularia* spp., which first appeared in the American province in PZ P5b [96], appears in Tethys (Plate 6, j) in the PZ P6b [32,111,114]. This west to east trans-Atlantic migration coincides with one of two global sea-level low stand in the early Ypresian (migration 5 in Figs 8, 13 and 14).

#### The Indo-Pacific Province

We found in our earlier studies of Paleogene rotaliids that migration from Tethys to the Indo-Pacific commonly occurred. Likewise, here we find examples of eastward migration in the Eocene of some **Alveolinidae** into the Indo-Pacific province after their first appearance in Tethys (Fig. 14). Thus, the first species of *Alveolina* appears in the late Ypresian (PZ P9; migration 6 on Fig. 15) in the Indo-Pacific province, compared with its occurrence at the base of Thanetian (PZ P4b) in the Tethys. The Indo-Pacific alveolinids of the Paleogene remained of lower diversity than those of their Tethyan ancestors. Their sizes were smaller (ranging between 7 and 10 mm [23]) and they showed less internal morphological variation. Only a few species have been reported from Indonesia, and many of them are of the *Alveolina oblonga* group of Hottinger [8]. The Indo-Pacific alveolinids range from the latest Ypresian (P9) to Bartonian [115]. Other species reported from the Indo-Pacific province include *Alveolina timorensis* Verbeek and *Alveolina ovicula* Nuttall [116].

**Figure 17:**
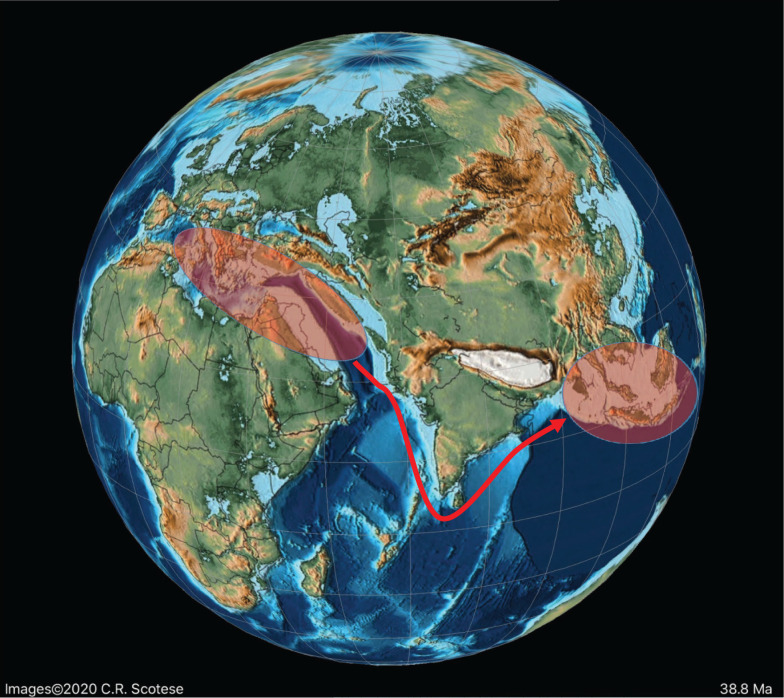
Schematic migration of alveolinoids during Late Paleogene, shown by red arrows, from the Tethyan province to the Indo-Pacific province.

*Borelis*, which first appeared in the Bartonian in Tethyan province, migrated towards the Indo-Pacific through the Tethyan corridor (Fig. 17), but did not appear in the Indo-Pacific province (migration event 9 in Fig. 8 [1,115,117]) before the Priabonian (PZ P15b; letter stage, Tb). For example, the Tethyan *Borelis vonderschmitti* (Schweighauser), which was widespread throughout the Middle–Late Eocene in the Western Tethys [104], appeared in the Late Eocene of the Indo-Pacific province [118]. The Tethyan Oligocene species of *B. inflata* are recorded also from the Oligocene–Miocene of Sarawak (Borneo) in the Indo-Pacific area [118,119]. A gradual stratigraphic succession of Indo-Pacific species marked its very slow evolution throughout the Oligocene.

Some Tethyan **Fabulariidae** forms also migrated into the Indo-Pacific province [120–122], where they are represented by *Lacazina* and *Lacazinella. Lacazina* reached the Indo-Pacific (migration 8 in Fig. 8) in the Bartonian (late P12a) and settled there by the late Eocene. *Lacazina* representatives are confined to the northern margin of the Indo-Pacific realm and are found only on an isolated platform of Moluccas [31]. *Lacazinella*, restricted to the southern part of the Tethyan province in the early Eocene (Somalia, Oman), first appears in the Lutetian in the Indo-Pacific province (migration 7 in Fig. 8) and in the Late Eocene (Papua New Guinea). This genus is recorded from Mindoro and Timor, and on remnants of the Australian craton in Indonesia [122]. *Lacazinella* did not survive the E–O extinction. *Neolacazopsis*, the representative of the Fabulariidae in the Middle to Late Eocene of Japan, evolved from *Lacazina* by developing turtleneck, bottle-like chamberlets and alveolar inner wall instead of arcuate chamberlets [123].

### The Neogene Alveolinoidea

Only **Alveolinidae** survived into the Neogene. The eastward migration towards the Indo-Pacific province along the Tethyan corridor previously seen in the Paleogene continued into the early Neogene. As species became geographically isolated, the alveolinid distribution showed a distinct provincialism. They colonised new areas in the Indo-Pacific and evolved similar but distinct parallel lineages, taking advantage of empty niches and optimal conditions.

#### The Tethyan/Mediterranean Province

In the Miocene, the Tethyan alveolinids were represented by species, such as *B. inflata*, *Borelis melo* and *Borelis curdica. Borelis inflata* is recorded as ranging from the Oligocene to the Miocene (Tortonian) of the Western Tethys [107,110,124], and Zakynthos in Greece [108,109]. *Borelis melo* with a spheroidal shape and relatively large proloculus (60–65 μm) has been reported from the Aquitanian to Messinian in the Tethyan province [105,106,125–127]. According to Bassi et al. [105], in *B. melo* the Y-shaped septula occur only in the adult growth stage, while in *B. curdica*, the proloculus is smaller (30–50 μm) and the Y-shaped septula occur in all whorls. These authors also postulated that all reliable records of *B. melo* and *B. curdica* are only from Western Tethys, and *B. curdica is* restricted to the Tethyan province. *Borelis melo* ranges from the Aquitanian to the earliest Messinian, while *B. curdica* ranges from the Burdigalian to the Tortonian [105,106,126,128–132]. For a comprehensive study and complete references to *Borelis* species, the reader is referred to Jones et al. [133] and Bassi et al. [105].

**Figure 18:**
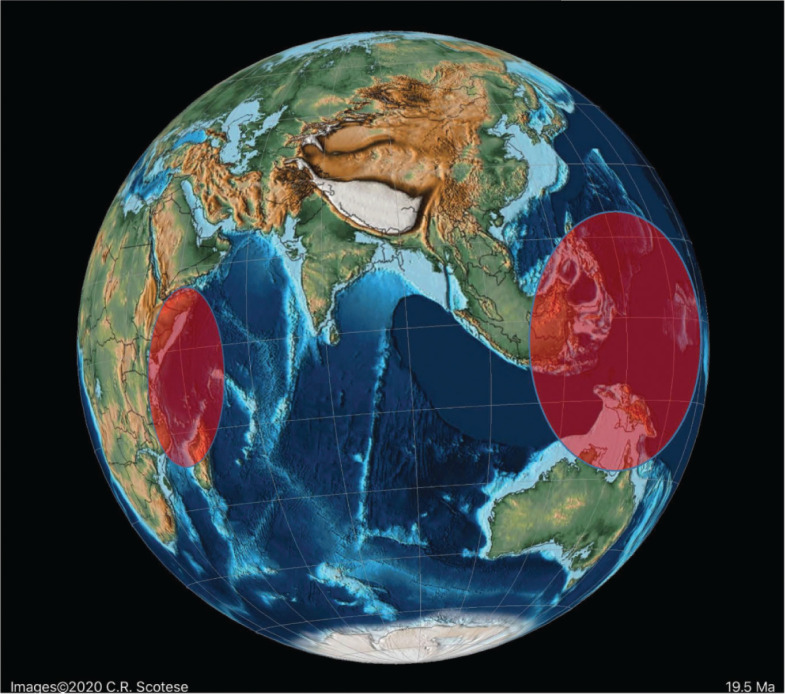
Schematic distribution of the reported alveolinoids during the Miocene in the Indo-Pacific province.

Tethyan alveolinid species became isolated from the Indo-Pacific by the final eastern closure of the Mediterranean basin (Fig. 18), and finally disappeared from the Mediterranean during the Messinian salinity crisis. However, living *Borelis* species have been reported from the shallow (less than 40 m) continental shelf off Israel [134]. Their re-occurrence in the Eastern Mediterranean with other LBF is attributed by Hyams et al. [134] either to migration from the Atlantic during warm periods of the Pleistocene or Holocene, or to modern migration from the Atlantic, or to modern (Lessepsian) migration from the Red Sea via the Suez Canal. Since the opening of the Suez Canal in 1869, the flow of Lessepsian species (i.e. Indo-Pacific species introduced into the Mediterranean Sea via the canal) has been continuous [135]. We suggest that a Lessepsian migration is the most likely way that *Borelis* has recently been introduced to the Eastern Mediterranean, as it is not found in the Western Mediterranean, which would be expected had the migration been from the Atlantic. Furthermore, Hyams et al. [134] figured solid, sub-ellipsoidal specimens morphologically similar to the Indo-Pacific *Borelis schlumbergeri* (Reichel). The Lessepsian origin of this finding is further supported by the fact that *B. schlumbergeri* (Reichel) is to be found in the Northern Bay of Safaga, in the Red sea, close to the southern entrance to the Suez Canal [136].

#### The Indo-Pacific Province

**Plate 7:**
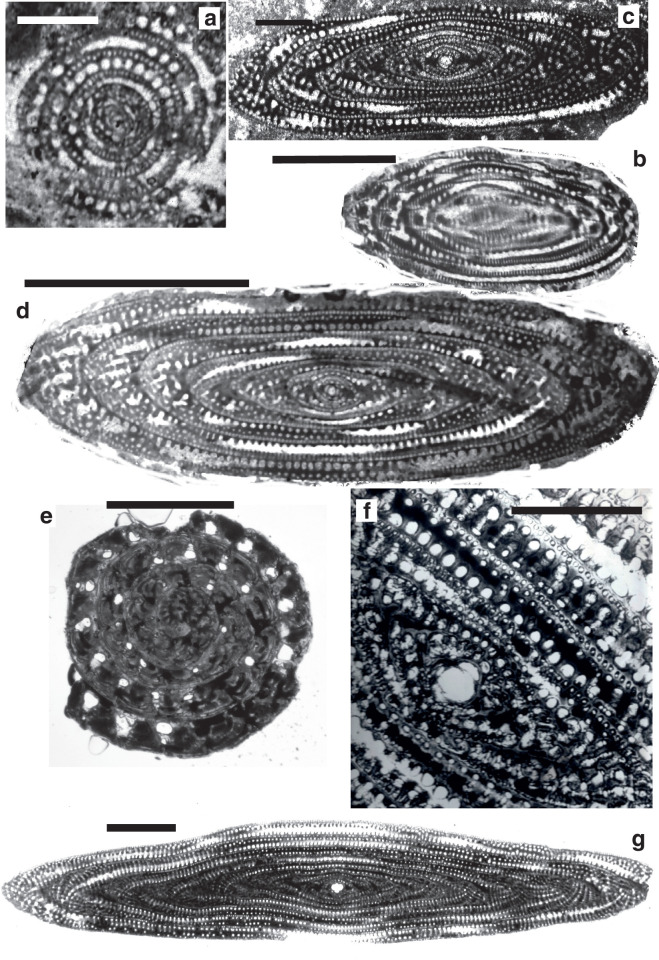
Scale bars: a–g = 1 mm. a. *Flosculinella globulosa* (Rutten), Early Miocene (lowermost Burdigalian), Indonesia, UCL coll. b. *Flosculinella bontangensis* (Rutten), late Early Miocene (Burdigalian, lower Tf1), Borneo, UCL coll. c. *Alveolinella praequoyi* Wonders and Adams, holotype, early Middle Miocene (upper Tf1–Tf2), Darai Limestone, Papua New Guinea, NHM P52658. d. *Alveolinella fennemai* Checchia-Rispoli, late Early Miocene (Burdigalian), Borneo, UCL coll. e–g. *Alveolinella quoyi* (d Orbigny), Holocene, e) Sekau, formation SEA coll., Royal Holloway; f) Port Moresby, Coral Sea, New Guinea, UCL coll; g) Pacific, UCL coll.

In contrast to the apparent isolation of Tethys forms from the Americas in the Middle and Late Paleogene, migration towards the Indo-Pacific continued in the Oligocene and Early Miocene through the Tethyan seaway (see [3–6]). In the late Langhian–early Serravallian (13–14 Ma), a time of several regressions, the short-lived marine reconnection between the Mediterranean and Indian Ocean again closed [137]. This final closure brought all migration towards the Indo-Pacific province to an end, and coincided with the onset of a global cooling period. By the time the East Antarctic Ice Sheet was established (12 Ma), the forereef LBF such as the miogypsinids and lepidocyclinids had become extinct or very rare in the Indo-Pacific sub-province [1], while the isolated backreef LBF in the Indo-Pacific developed new survival characters. The small primitive *Borelis* in Middle to Late Miocene shallow-water settings became rare and was gradually replaced by forms with more evolved characters [1,27], such as *Flosculinella* (Plate 7, a–b) with double rows of chamberlets on the floor of each chamber, and the elongated fusiform *Alveolinella* with several rows of chamberlets in axial section. The superposed regular layers of chamberlets separated by floors in *Alveolinella* (Plate 7, c–g) are analogous structures found in the mid-Cretaceous *Praealveolina*, and are different from the irregular, tubular, supplementary chamberlets present in the thickened basal layers of the Paleogene *Alveolina* [67]. They re-appear, through convergent evolution (homoplasy), millions of years after their earlier extinction, as they are selected for by the changed ecological conditions developed in the Middle Miocene.

Over the Neogene Period, Indo-Pacific alveolinids underwent a very slow evolution, but notably include *Borelis pygmaeus* (which persists to the top of the Te stage [1,27]). This species is recorded from Oligocene to Lower Miocene deposits [23,27,118,119], but is suddenly replaced by a more advanced form *Flosculinella bontangensis* (Plate 7, b), with an incremental increase in the length of the test from pole to pole, changing from globular, to ovoid, to elongated spindle-shaped, and with an equally incremental increase in the number of secondary chamberlets (‘mansardes’) from zero in *Borelis* through to one in *Flosculinella* to several rows of chamberlets in *Alveolinella.* Parallel lineages have each developed with the acquisition of an additional row of chamberlets in the *Borelis–Flosculinella* lineage. This must have occurred at least twice during Te–Tf interval (Early–Middle Miocene). An example of one of these lineages is seen with the appearance of *Flosculinella reicheli* within the Early Miocene (upper Te) and of *F. bontangensis* (Plate 7, b) in the early Middle Miocene (just at the top of the Te stage). The latter gave rise to *Alveolinella praequoyi* (Plate 7, c) in the Serravallian, at the base of Tf2 stage. *Alveolinella praequoyi* has early whorls akin to *F. bontangensis*, but in the latter the chamberlets of each whorl are covered by at least two layers of smaller chamberlets. In *Alveolinella quoyi* (Fig. 9; Plate 7, e–g), in the Tf3 stage, all of the whorls have a multiple layer of chamberlets. *Flosculinella bontangensis* grades into forms of *Alveolinella* at about the same time as the onset of the global cooling. *Alveolinella* today appear to be confined to normal shallow marine (down to depths of 80 m), clear, well-oxygenated, tropical and subtropical waters [39,138].

Different parallel lineages of *Borelis* spp. evolved in the Indo-Pacific area. Those with Y-shaped septula such as *B. pygmaeus* are recorded in the Indo-Pacific from the Rupelian to latest Burdigalian (uppermost upper Te [27]), while other lineages without the Y-shaped septula in the juvenile stage, such as *B. schlumbergeri*, are recorded from the Tortonian to Holocene [105,139,140].

Bassi et al. [105] assigned the Indo-Pacific Oligocene to Early Miocene species of *Borelis primitiva* (described from an Upper Oligocene limestone from Eniwetok [141]), *Borelis globose, Borelis boninensis* (described from the Rupelian of east Mindanao and from the the Bonin Islands [118,142]), and the specimen of *B. melo* (reported by BouDagher-Fadel [1] from the Serravallian of Australia), to *Borelis pulchra*. They based their conclusion on the absence of Y-shaped septula and the proloculus size of *B. pulchra*. In our opinion, however, these species all have specific characteristics and are separated by time and space from *B. pulchra.* We suggest, therefore, that they should still be treated as separate species.

*Borelis inflata*, which first appeared in the Oligocene of Tethys, migrated rapidly into the Indo-Pacific province, and has been described from the Oligocene to Lower Miocene of Sarawak (Borneo) in the Indo-Pacific area [118,119].

#### The American Province

The alveolinids were absent from the Neogene of the American province before the early Piacenzian closure of the Central America seaway (Figs 13 and 16). Today, they are represented by *B. pulchra* (d’Orbigny), which is recorded from present-day sands of Cuba, Florida and from Jarvis Island [105,138,143–145].

There are no records for this species from the Mediterranean, nor would we contend from the Indo-Pacific (see above). Bassi et al. [105], however, suggest that Indo-Pacific forms are synonyms of *B. pulchra* and proposed that there was a migration eastwards from the Indo-Pacific across the Pacific and through the Central America Seaway, before its closure in the Pliocene (Fig. 13). However, despite being strong advocates for the dispersal of LBFs by trans-oceanic migration, because in our opinion the Pacific species are distinct from the American *B. pulchra*, we favour the suggestion that *B. pulchra* is another example of homoplasy, just as we suggest that the Middle Eocene Tethyan origin of *Borelis* was distinct from that of the early Paleogene American forms.

## Discussion

The synthesis described here of the alveolinoids, over an expanded, globally comprehensive geographic area and stratigraphic range, has enabled a more coherent view of their phylogenetic and palaeogeographic evolution to be established, and provides an update to the description presented in BouDagher-Fadel [1]. Two significant characteristics of their evolution and development will be discussed below in further detail, namely those of homoplasy, and of provincialism punctuated by trans-oceanic migration.

The traits of convergent evolution exhibited by the alveolinoids fall into two distinct types: those in which gross external morphology of forms echo the shape of previous LBF forms, and those in which the internal test morphology is reproduced in forms that are not directly consanguineous. As noted above, an extreme example of ecologically driven convergent evolution involving forms from different orders is exhibited in the external appearance of alveolinoids that closely resemble the planispiral, fusiform fusulinides of the late Paleozoic.

More confusingly from the taxonomic and phylogenetic viewpoint, however, are the second type of homoplasy, where due to a predisposition to repeated genetic mutations, forms with the same or similar generic characteristics repeatedly appear within a family over time. When these characteristics are similar but not identical, they can lead to the misidentification of forms, as for example, in the rhapydioninids, where some Cretaceous Tethyan *Murciella* have been misidentified as *Raadshoovenia*. As noted above, true *Raadshoovenia* appeared only in the Paleogene of the American, and can be characterised by having a streptospiral nepiont in both megalospheric and microspheric generations, and by being smaller than *Murciella*.

Another example of homoplasy is one that gives rise to very similar forms within the same family, but which are separated by a larger stratigraphic interval. So, for example, following their Cretaceous predecessors, Paleogene alveolinids re-appeared from *Pseudonummuloculina*, giving rise to the *Alveolina* lineage. These Paleogene forms are morphologically convergent with the fusiform/globular alveolinids of the mid-Cretaceous characterised by the *Praealveolina* group. There are generic differences to the detailed test structure of these two lineages, as well as a considerable gap of about 20 Ma in the fossil record, so there is no doubt that they represent distinct, homoplasic stages of evolution within the Alveolinidae. The same is not true, however, for the Late Cretaceous and Early Paleogene fabulariids. Here, the Paleocene *Periloculina* lineage (including *Pseudolacazina* and *Lacazina*) echoed the Cretaceous forms so closely that they are generically indistinguishable. There is however a gap of over 6 Ma between the K–P boundary and the first Paleogene occurrence of *Periloculina* in the Selandian. These Paleogene genera could be considered to be Lazarus taxa, which Wignall and Benton [146] explain thus, ‘At times of biotic crisis many taxa go extinct, but others only temporarily disappeared from the fossil record, often for intervals measured in millions of years, before reappearing unchanged.’ Wignall and Benton [146] suggest that the reappearance of Lazarus taxa in the fossil record probably reflects the rebound after a period of extreme rarity during the aftermath of a global extinction. Here, however, we prefer another explanation, namely that, as with the *Alveolina* lineage, the Paleogene *Periloculina* lineage is an example of homoplasic re-evolution from a small, simple form that survived the K–P extinction. However, unlike *Alveolina* we suggest that, for the Paleogene *Periloculina* lineage, the homoplasy produced forms so close to the previous Cretaceous forms as to be generically indistinguishable.

A further example of a form that could be a Lazarus taxa or an example of homoplasy, is given by *Borelis*. The American *Borelis* is reported from the Middle Paleocene to the Early Eocene (PZ P–P9), while the first occurrence of the Tethyan *Borelis* is in the Bartonian of Western Tethys. These occurrences are separated by 6.6 Ma in the geological column, and by an ocean. American *Borelis* has aligned septula and pre-septal passages, and they are in general smaller in size than those of the Tethyan *Borelis*. This, and the challenge of the concept of ‘the rebound after a period of extreme rarity’ of a form into a province displaced from its original realm, leads us to propose that American *Borelis* and Tethyan *Borelis* are likely also to be examples of homoplasy. This potential tension between the concepts of Lazarus taxa and of homoplasy is worthy of further exploration elsewhere.

We now turn to the second notable feature of the development of the Alveolinoidea, namely their distinct global pattern of provincialism and the episodes of trans-oceanic migration, similar to behaviour previously observed in other LBFs (e.g. [3–6,147,148]). The three main alveolinoid provinces were those of the Americas, Tethys and the Indo-Pacific. We have previously demonstrated that the Cretaceous agglutinated orbitolinids originated in Tethys, but underwent periodic westward trans-oceanic migration to the Americas during periods of global sea-level low stands [2]. In this study, we have confirmed that the Tethyan province also remained the palaeogeographic centre for the porcelaneous alveolinids, and that they inhabited similar ecological habitats throughout the Cretaceous and Paleogene. It has been inferred here for the first time that a westward palaeogeographic migration of some alveolinid genera also occurred in the mid-Cretaceous from the Tethys to the Americas (migrations 1–3, Fig. 11), while the Late Cretaceous alveolinids remained mainly isolated (migration 4, Fig. 11). On the other hand, in the Paleogene we saw limited eastward migration from the American province towards Tethys (*Fabularia* in the Ypresian, PZ P6b; migration 5, Fig. 15). On the other hand, migrations from Tethys towards the Indo-Pacific region continued till the closure of the Tethyan corridor and the isolation of the Mediterranean in the Serravallian (migrations 6–9, Fig. 8). As noted, we have shown that most cosmopolitan alveolinoids appeared in Tethys before migrating to other provinces. Likewise, we have seen that once established in the American and Indo-Pacific provinces, local provincial forms evolved, indicating that they were periodically effectively isolated from the Tethyan province. These characteristics have been observed in previous studies of Mesozoic [2] and Cenozoic LBF, specifically the lepidocyclinids [3], the miogypsinids [4], the nummulitoids [5,149] and the orthophragminids [6,150]. The periods of migration from one province to another were often followed by subsequent isolation and development of local provincial lineages. These migrations were often associated with major sea-level regressions, while the subsequent provincial isolation coincided with global sea-level transgressions. This study has, therefore, further tested the migration hypothesis highlighted by Pignatti [7], and has confirmed the wider application of the ideas initially put forward by BouDagher-Fadel and Price [2–6].

## Conclusions

We have resynthesised the literature on the occurrence and distribution of the alveolinoids, and augmented this information with observations on new materials from the Americas, Europe, the Middle East, Tibet and South-East Asia. We have produced a global correlation and integration of the occurrences and stratigraphic ranges of members of the three alveolinoid families, namely the Alveolinidae, the Fabulariidae and the Rhapydioninidae, based upon the planktonic foraminiferal zonal scheme of BouDagher-Fadel [1,24]. In the Cretaceous, alveolinoids are found in the Tethyan and American provinces, while of the later Cenozoic forms, Alveolinidae and Fabulariidae are found in three provinces (Tethys, American and the Indo-Pacific), while the Rhapydioninidae are constrained to the Americas.

Alveolinoids characteristically exhibit convergent evolution, with the repeated re-occurrence of certain morphological features. This homoplasy is predominantly of two types, one which recapitulates the gross external morphology of forms, and one in which the internal test morphology is also reproduced, even to the level of regenerating forms with the same generic characteristics.

We have shown that no alveolinoid survived the K–P extinction, but that members of all three families re-appeared from small miliolids that were resilient enough to pass into the Paleogene. Specifically, we have concluded that:

- Cretaceous Tethyan *Murciella* has on occasion been misidentified as a *Raadshoovenia*, which in fact is an American Paleocene form.
- *Fabularia* originated in the Americas at the beginning of the Ypresian, but soon thereafter migrated to Tethys.
- There is a gap of over 6 Ma between the last occurrence of *Periloculina, Pseudolacazina* and *Lacazina* in the Cretaceous and their re-appearance in the Paleogene, and that these taxa are most likely to be examples of homoplasic evolution from a small, simple form that survived the K–P extinction.
- The 6.6 Ma gap between American *Borelis* and Tethyan *Borelis* suggests that they are likely also to be examples of homoplasy.

Finally, our previously developed hypothesis that periodic, sea-level lows enable inter-provincial migration of LBF is strongly supported, and that the behaviour and directions of migration of the alveolinoids is resonant with those of the orbitolinids, the orthophragminids, the nummulitoids, the miogypsinids and the lepidocyclinids. Migration occurred from East to West in the Cretaceous, but was dominated by migration from West to East in the Cenozoic.

## Data Availability

All data generated or analysed during this study are included in this published article (and its supplementary information files).
